# Artificial Nervous Systems

**DOI:** 10.1002/advs.202511478

**Published:** 2025-09-14

**Authors:** Lu Yang, Qingwen Cheng, Yue Li, Ning Wu, Zhipeng Xu, Wentao Xu

**Affiliations:** ^1^ Institute of Photoelectronic Thin Film Devices and Technology Key Laboratory of Photoelectronic Thin Film Devices and Technology of Tianjin College of Electronic Information and Optical Engineering Engineering Research Center of Thin Film Photoelectronic Technology of Ministry of Education Academy for Advanced Interdisciplinary Studies Nankai University Tianjin 300350 China; ^2^ Shenzhen Research Institute of Nankai University Shenzhen 518000 China

**Keywords:** artificial nervous system, multimodal perception, neural signal processing, neuromorphic device, synaptic device

## Abstract

Electronic devices and systems that can emulate the complex cognitive abilities of nervous systems have been an important research topic in recent years. Artificial nervous systems with multimodal perception, neural signal processing, and reflex‐driven functionalities have been established by integrating multimodal sensors, neuromorphic synaptic devices, and effector units. The important value of artificial nervous systems is mainly embodied in bioinspired information processing, transmission, and environmental adaptation aspects. However, since this field is still in its early stages, more exploration is needed to realize artificial nervous systems with autonomous adaptability, intelligent feedback, and bio‐interfacing applications. This review provides a comprehensive overview of recent advancements, spanning from the fundamental device structure and mechanism of synaptic devices to bio‐inspired artificial nervous systems. Finally, a brief perspective of this research field is provided.

## Graphical abstract

By drawing inspiration from biological neural hierarchies and the working mechanisms of plasticity, researchers have constructed a series of bionic devices, including sensory devices, synapse devices, and artificial neural systems. They committed to simulating and surpassing the biological information processing function, thereby realizing the perception, cognitive, and memory processing of complex external stimulus information, and outputting interactive information in real‐time.

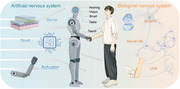

## 1 Introduction

**Figure 1:**
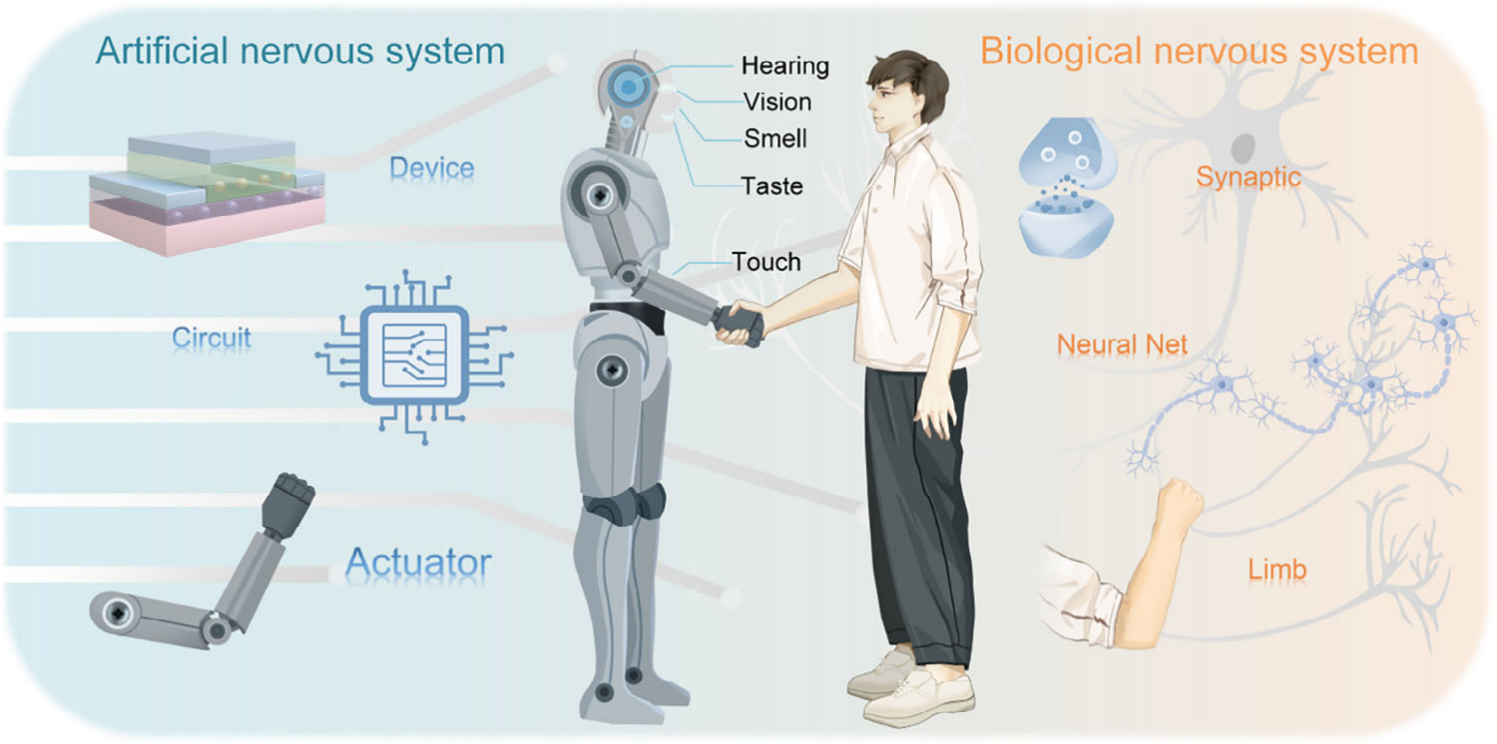
The biological nervous system and the artificial nervous system.

The human nervous system processes and transmits information through neurons and synapses, operating in a distributed, parallel, and event‐driven mode.^[^ ^1^, ^2^ ^]^ It is capable of efficiently and intelligently sensing, processing, and responding to complex external stimuli, enabling real‐time interaction and adaptive regulation. By drawing inspiration from biological neural hierarchies and the working mechanisms of plasticity, combined with continuous breakthroughs in the field of new materials and the advent of artificial intelligence,^[^ ^3^ ^]^ researchers have constructed a series of bionic devices, including sensory devices,^[^ ^4^, ^5^, ^6^ ^]^ synapse devices,^[^ ^7^, ^8^, ^9^, ^10^, ^11^ ^]^ and artificial neural reflex arc systems.^[^ ^12^ ^]^ They committed to simulating and surpassing the biological information processing function, thereby realizing the perception, cognitive, and memory processing of complex external stimulus information, and outputting interactive information in real‐time (**Figure** **1**).^[^ ^13^ ^]^

**Figure 2:**
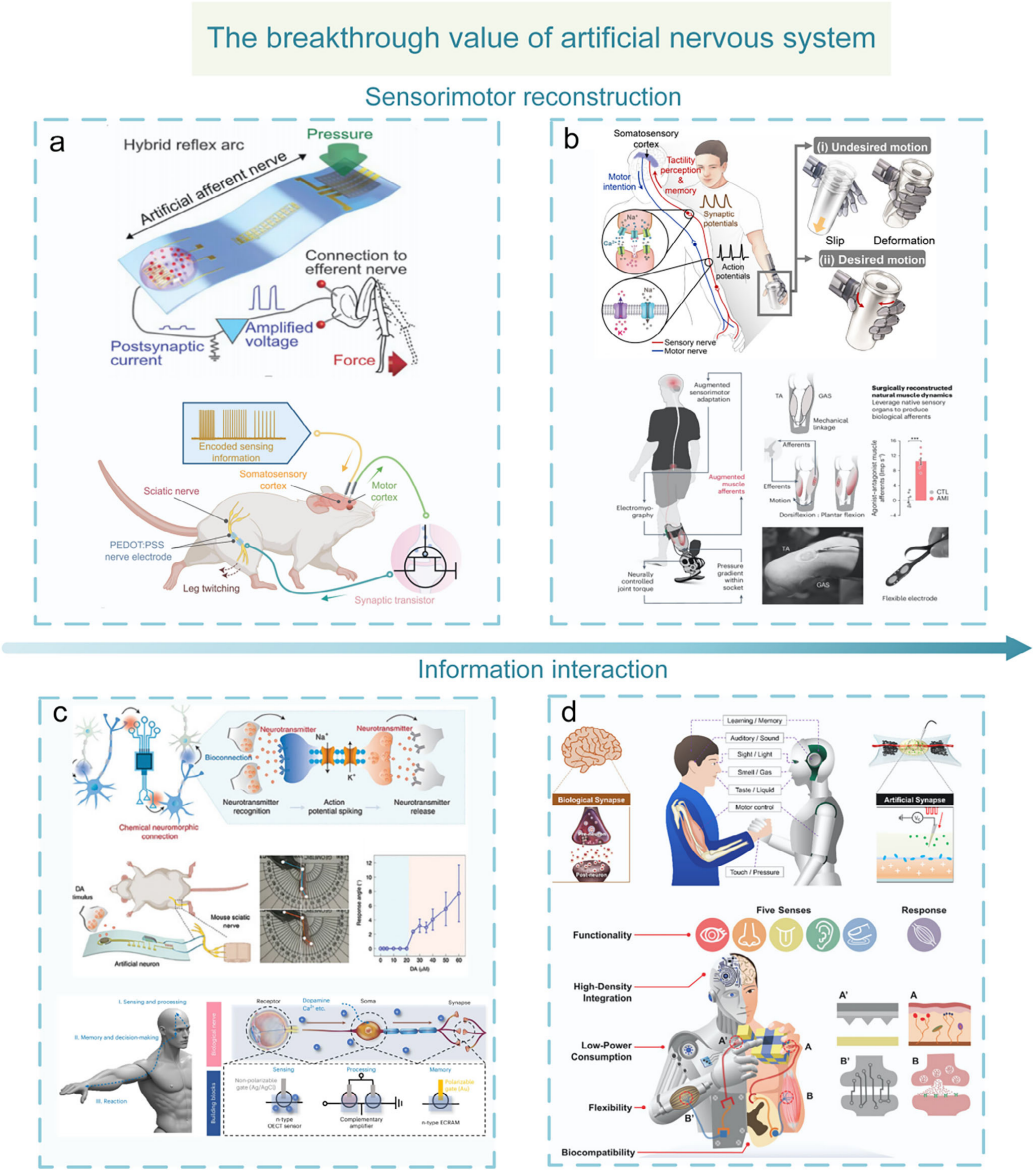
The breakthrough value of an artificial nervous system. a,b) The level of sensorimotor function reconstruction. Sensorimotor function simulation and substitution.^[^ ^14^, ^15^ ^]^ Copyright 2018, The American Association for the Advancement of Science. Sensorimotor function reconstruction.^[^ ^14^, ^15^ ^]^ Copyright 2023, The American Association for the Advancement of Science. Copyright 2024, The Author(s). c,d) The level of information interaction. Neurotransmitter detection.^[^ ^18^, ^19^ ^]^ Copyright 2022, The Author(s), under exclusive licence to Springer Nature Limited. Human–machine interaction.^[^ ^20^, ^21^ ^]^ Copyright 2019, American Chemical Society. Copyright 2019 WILEY‐VCH Verlag GmbH & Co. KGaA, Weinheim.

So far, artificial nervous systems have proven their applicability to neuromorphic image recognition, neuromorphic prostheses, neuromorphic soft robots, and neuromorphic biohybrid systems. It has also shown potential application prospects in the fields of biological neural function emulation and substitution,^[^ ^14^, ^15^ ^]^ prosthetic limb control,^[^ ^16^, ^17^ ^]^ intelligent health monitoring,^[^ ^18^, ^19^ ^]^ and information interaction (**Figure** **2**).^[^ ^20^, ^21^ ^]^ Artificial nervous systems are valuable in two strategic aspects. One is at the level of sensorimotor function reconstruction.^[^ ^22^ ^]^The advancement in flexible biomimetic materials has enabled the artificial nervous system to directly connect to biological nerves, establishing a millisecond response “bio‐interactive interface.” This system has successfully reconstructed sensorimotor functions of spinal cord‐injured rats,^[^ ^23^ ^]^ and realized the decoding of artificial retinal signals.^[^ ^24^ ^]^ A hardware‐algorithm co‐optimization architecture is created through integrating artificial nervous system, bionic materials, and artificial intelligence technology, which can reduced the system response latency to 1.8 ms,^[^ ^25^ ^]^ and the accuracy rate of clinical prostheses in performing complex movements can be greatly improved, providing innovative diagnosis and treatment solutions for 3 billion individuals with neurological disorders and 1.3 billion with limb disabilities. Another is at the level of neural information interaction. Artificial nervous systems can convert multimodal stimuli, such as pressure, temperature, and light, into brain‐like compatible signals, realizing biologically multimodal perception and information cognition. This hardware‐level compatible architecture can greatly simplify the complexity of human–machine interaction algorithms.

However, since this frontier field is still in its infancy, to achieve an artificial nervous system with intelligent feedback and practical clinical applications, it is necessary to comprehensively simulate and reproduce the complex information cognitive processing mechanism of biological systems, and optimize the existing research system from multiple dimensions of “materials–devices–systems.” Therefore, this review first introduces the mechanisms of information perception and processing in biological systems. Then it discusses the classification and research progress of information processing and transmission artificial synaptic devices, which are the core units for various types of sensory and motor neurons. Next, it reviews the progress in bio‐inspired artificial neural systems. Finally, it summarizes current applications and provides an outlook on future development trends.

## 2 Biological Neural System and Synapse

**Figure 3:**
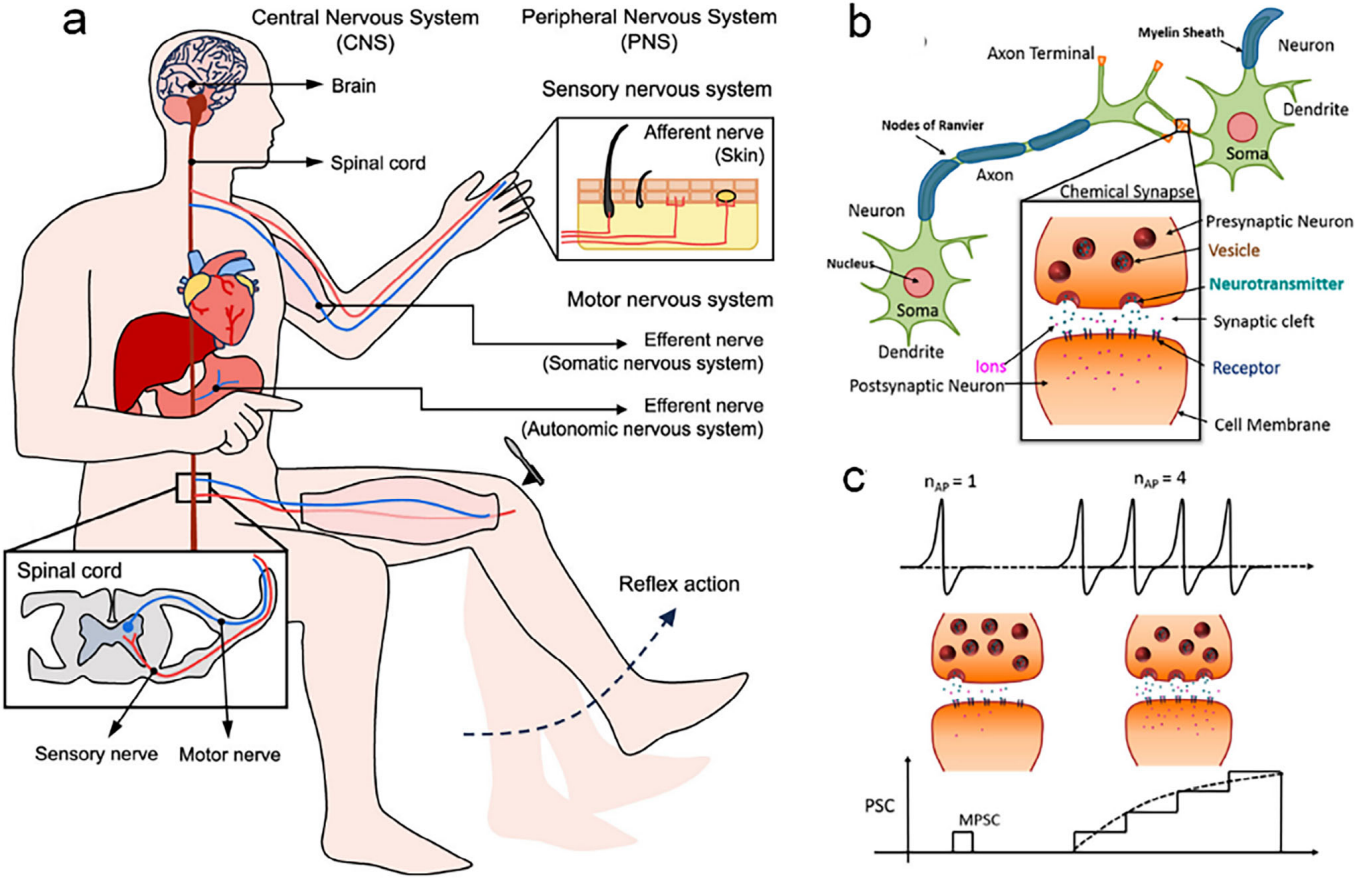
Biological nervous system. a) The central and peripheral nervous systems.^[^ ^26^ ^]^ Copyright 2025, American Chemical Society. b) Structure of biological neurons and synapses. c) The transformation process from STP to LTP.^[^ ^29^ ^]^ Copyright 2017, American Chemical Society.

It is widely accepted that the human nervous system is comprised of two distinct components: the peripheral nervous system (PNS) and the central nervous system (CNS). The CNS is the body's primary unit with the world of information processing. It is comprised of the spinal cord and brain, and its primary functions are the transmission, storage, and processing of information. The CNS is the neural basis for memory. The PNS consists of nervous fibers and neuronal cell bodies located outside the CNS. It is functionally subdivided into the motor and sensory nervous systems. The sensory nervous system consists of afferent nerves, which connect peripheral receptors to the CNS. The motor nervous system consists of efferent nerves, which are responsible for transmitting information processed by the CNS and controlling muscle contraction and movement (**Figure** **3**a).^[^ ^26^ ^]^

For the complete reflex system of the human body, external stimuli (such as force, light, and heat) are first preprocessed by the sensory system, then encoded and transmitted to the brain for processing. Finally, the processed information is output through motor nerves to control effectors (such as muscles and glands) to complete specific actions. In this process, the sensory system plays a crucial role as the intermediary between the brain and the external environment. The human sensory system primarily includes five major types: vision, hearing, touch, smell, and taste, with different sensory receptors playing important roles in information perception. By altering the connection strength between neurons, the multilevel nervous system collaboratively performs real‐time detection, transmission, integration, cognition, and processing of information. Throughout the whole process, the CNS transmits sensory information and produces related cognition and memory, enabling organisms to accurately perceive external information and control effectors to make corresponding responses, thereby completing a series of reflex activities. In real complex events, compared to single‐sensory modalities, the multi‐sensory integration mode occupies a major position, which integrates parallel information from different sensory pathways into a comprehensive consciousness. And eliminating uncertainties in distinguishing external stimuli through parallel transmission and collaborative processing mode, so that the organisms can exhibit exceptional immediacy and accuracy in information processing.^[^ ^27^ ^]^

The biological nervous system is comprised of a large number of neurons (≈10^12^), and every neuron is connected to other neurons through thousands of synapses. Neurons and synapses are connected in different ways to build a complex neural network system, which ensures the human body's efficient parallel computational ability and strong adaptive ability.^[^ ^1^ ^]^ Biological systems communicate based on ions and small molecules, while silicon‐based electronic devices rely on electronics.^[^ ^28^ ^]^ The development of biologically similar ion transport systems can directly mimic biological functions, thus effectively bridging the gap between artificial intelligence and biological systems. Therefore, constructing ion‐transporting bionic devices is a major research focus. Neurons are the fundamental units of the nervous system, which consist of dendrites, cell bodies, and axons. Dendrites are responsible for receiving signals from the previous neuron and passing them to the postsynaptic neuron through the axon and synapse. Synapses are generally formed between the axon of the anterior neuron and the cell body or dendrites of the posterior neuron, and are responsible for storing and transmitting information between the various levels of neurons (Figure 3b).^[^ ^29^ ^]^ The holes on the surface of neurons act as ion channels for synaptic information transfer. The channel forms an ion concentration difference through cellular signaling in response to external stimuli, and signal transmission is accomplished between anterior and posterior neurons via vesicles. And leads to electrically active behavior in neurons, realizing information, memory, and processing.

The input signals from preneuron axons can change the connection strength (synaptic weights) between neurons. Synaptic weights can be enhanced or inhibited based on the type of input stimulus, directly influencing signal transmission efficiency and enabling diverse functions. This property is known as synaptic plasticity, which plays a crucial role in neural signaling and underlies the brain's functions of memory, cognition, computation, and learning.^[^ ^30^, ^31^ ^]^ Plasticity can be categorized into short‐term plasticity (STP) and long‐term plasticity (LTP) based on the length of memory time, and can be switched by changing the frequency and duration of learning (Figure 3c).^[^ ^29^ ^]^ Plasticity is essential for organisms to learn and remember. In addition to LTP and STP, biological synapses also exhibit associative learning, spike frequency‐dependent plasticity (SFDP), spike timing‐dependent plasticity (STDP), and spike number‐dependent plasticity (SNDP).^[^ ^32^ ^]^ These various forms of plasticity are the foundation and prerequisite for the brain to accomplish information perception, processing, transformation, and transmission.

Neurons serve as the fundamental building blocks, and synapses act as the bridge for information transmission between neuronal groups, forming the basis for information perception and cognition. Therefore, in order to comprehensively simulate the functions of the CNS and PNS, artificial synaptic electronic devices are used as the basis to simulate the plastic functions of biological synapses to achieve learning and memory. Based on the complex dynamic plasticity switching and high expandability of synaptic devices, they are used as information processing units to further construct complete artificial neural systems, achieving biological nerve‐like information perception, processing, and motion regulation.

## 3 Artificial Synaptic Device

Neuromorphic electronic devices were proposed in the 1980s with the aim of building electronic devices with a similar structure, function, and plasticity to biological neural networks. As a typical representative of neuromorphic electronic devices, artificial synaptic devices have been widely studied due to their significant advantages in information transmission and processing, attributed to their nonlinear conductance modulation characteristics under different modes of voltage stimuli (amplitude, frequency). In 2008, Hewlett‐Packard Company (HP) developed a two‐terminal synaptic device (memristor) with resistance and memory characteristics by using titanium dioxide (TiO_2_) nanofilm structure. This device is a sandwich structure composed of Pt/TiO_2_/Pt.^[^ ^33^ ^]^ Based on this, Jo et al. constructed a memristor based on silver‐doped amorphous silicon, which can precisely adjust the multilevel conductance state. This device achieved long‐term potentiation/depression (LTP/LTD) functions similar to biological synapses.^[^ ^34^ ^]^ Subsequent studies have been devoted to simulating and surpassing the important functions of biological synapses, such as plasticity through structural design and different material choices. The core working mechanism of synaptic devices mainly causes changes in resistance or conductance through ion migration, electron migration, and phase transitions, thereby generating memory effects. Therefore, on a macroscopic level, based on their working principles, synaptic devices can be roughly classified into ion‐migrating, electron‐migrating, and phase‐changing devices. From the perspective of structure, two‐terminal memristors and three‐terminal transistors are currently the most widely studied synaptic devices.^[^ ^35^ ^]^ These synaptic devices are all capable of simulating synaptic plasticity. Two‐terminal devices exhibit advantages in power consumption and size, and the simplicity in structure offers great potential for high‐density integration.^[^ ^36^ ^]^ However, their simplistic structures limit the ability to concurrently process and transmit signals. In contrast, three‐terminal synaptic transistor devices achieve simultaneous signal processing and transmission through gate modulation.^[^ ^37^ ^]^

### 3.1 Two‐Terminal Synaptic Device

**Figure 4:**
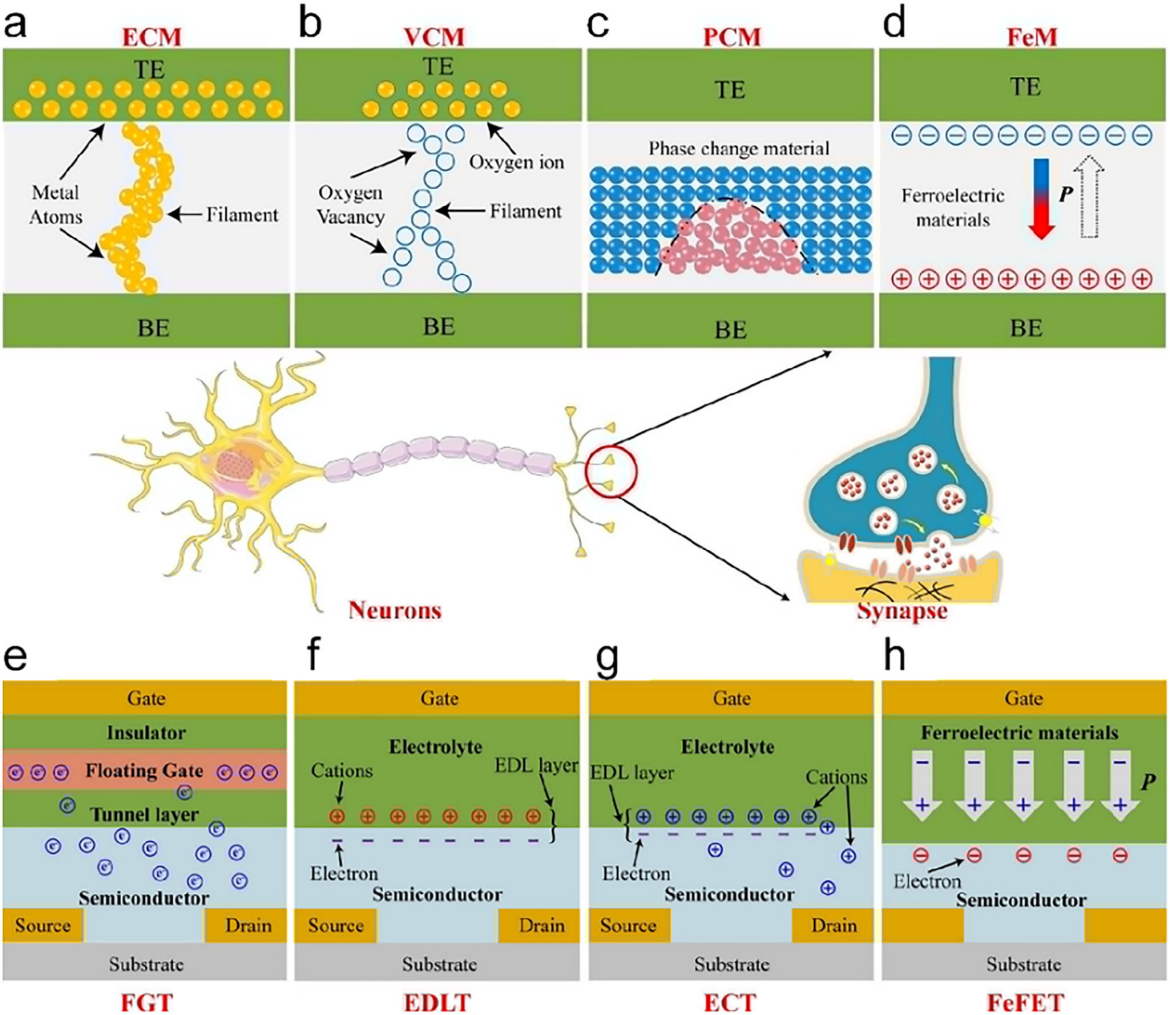
Structure diagrams of different types of two‐ and three‐terminal artificial synaptic devices, based on the operation principles. a–d) Diagrams of two‐terminal synaptic devices: ECM (rely on the migration and redox reactions of metal ions under voltage conditions), VCM (rely on valence changes of cations and the migration of oxygen anions), PCM (rely on a reversible phase transition from the amorphous to the crystalline state through a joule heating process), FEM (rely on the spontaneous polarization reversal of ferroelectric materials under an applied electric field), respectively. e–h) FGT (rely on the electronic charge can migrate to the floating gate and be captured under the effect of gate voltage regulation), EDLT (rely on the ion migration in the gate dielectric layer and channel layer under gate voltage), ECT (rely on the ion migration in the gate dielectric and the channel with redox properties), FeFET (rely on changing the ferroelectric material's polarization state), respectively.^[^ ^35^ ^]^ Copyright 2021, American Chemical Society.

Two‐terminal synaptic devices generally comprise top and bottom electrodes, with a functional layer positioned between them. The synaptic plasticity is realized through changes in the conductance of the functional layer. The top and bottom electrodes correspond to the presynaptic and postsynaptic membranes, respectively, and the functional channel layer corresponds to the intermediate synaptic cleft. According to the current research progress, two‐terminal synaptic devices can be roughly categorized by their working mechanisms into electrochemical metallization mechanism (ECM), valence change mechanism (VCM), phase‐change mechanism (PCM), and ferroelectric mechanism (FeM) (**Figure** **4**a–d).^[^ ^35^, ^38^, ^39^ ^]^

ECM‐based two‐terminal synaptic devices exhibit memory switching characteristics due to the migration and redox reactions of metal ions under voltage conditions.^[^ ^40^ ^]^ This type of device typically adopts a structure of “active electrode/solid electrolyte/inert electrode” (Figure 4a). The functional layer can adopt metal oxide materials, e.g., silver sulphide (Ag_2_S),^[^ ^41^, ^42^ ^]^ zinc oxide (ZnO),^[^ ^43^ ^]^ and copper(I) sulfide (Cu_2_S).^[^ ^44^ ^]^ Ohno et al. successfully constructed a device based on Ag_2_S that simultaneously simulated the STP and LTP functions of biological synapses.^[^ ^41^, ^42^ ^]^ The regulation of synaptic weights (conductance) can be controlled by adjusting the frequency and intensity of the applied electrical pulses. Besides, organic polymers.^[^ ^45^ ^]^ perovskite,^[^ ^46^ ^]^ and 2D materials.^[^ ^47^ ^]^ Xu et al. fabricated an organometalide perovskite synaptic device.^[^ ^46^ ^]^ Due to the presence of ion migration in the ionic perovskite matrix, they successfully emulated excitatory postsynaptic currents, paired‐pulse facilitation, STP, and LTP. ECM‐based synaptic devices can operate at low voltages and have a high switching ratio.

VCM‐based devices rely on valence changes of cations and the migration of oxygen anions to achieve their functionality.^[^ ^48^ ^]^ This type of device is typically composed of a functional layer and inert metal electrodes (Figure 4b). The functional layer can adopt ferroelectric polyvinylidene–trifluoroethylene copolymer (P(VDF–TrFE),^[^ ^49^ ^]^ poly 3, 4‐ethylenedioxythiophene polystyrene sulfonate (PEDOT:PSS),^[^ ^50^ ^]^ and polymethyl methacrylate (PMMA).^[^ ^51^ ^]^ hafnium oxide (HfO_x_),^[^ ^52^ ^]^ molybdenum disulfide with oxygen substitution (MoS_2_−_x_O_x_),^[^ ^53^ ^]^ molybdenum disulfide (MoS_2_).^[^ ^54^ ^]^ The resistance switch characteristics of 2D materials have attracted extensive attention. Li et al. constructed a two‐terminal synaptic device containing multilayer structures (Al/MoS_2_/polycrystalline silicon) based on the 2D material MoS_2_.^[^ ^54^ ^]^ The device demonstrates on/off ratio ≈10^4^, cycle stability reliability ≈10^3^, and retention time ≈10^4^ s. The I–V scanning results show that the Poole‐Frenkel effect dominates in the MoS_2_‐based device. The trap‐assisted tunneling transition effect and Schottky emission lead to a high resistance state (HRS) and a low resistance state (LRS), respectively. Common conduction mechanisms of VCM‐based synaptic devices include Schottky emission, ohmic conduction, trap‐assisted tunneling, space charge limited conduction, Poole‐Frenkel emission, and hopping conduction.^[^ ^35^ ^]^ Similar to ECM‐based devices, VCM‐based devices can operate at low voltages.

PCM‐based devices realize a reversible phase transition from the amorphous to the crystalline state through a joule heating process. This type of device is typically composed of two electrodes and a phase change material layer (Figure 4c). Kuzum et al. proposed and verified an artificial synaptic device based on chalcogenide glass (Ge_2_Sb_2_Te_5_).^[^ ^55^ ^]^ The Ge_2_Sb_2_Te_5_ undergoes reversible and precise proportional changes between crystalline and amorphous states through electrical pulses, thereby achieving continuous and multilevel regulation of its electrical conductivity. At present, a variety of high‐quality phase change materials have been developed for the functional layer, e.g., germanium tellurium (GeTe),^[^ ^56^ ^]^ silicon–antimony–tellurium (Si–Sb–Te),^[^ ^57^ ^]^ nickel oxide‌ (NiO_x_),^[^ ^58^ ^]^ strontium iron oxide (SrFeO_x_),^[^ ^59^ ^]^ etc. Su et al. adopted SrFeO_x_ as the functional layer and used electron beam evaporation to form Au electrodes.^[^ ^59^ ^]^ The Au electrode contact can be improved through the transfer process, which can effectively increase the on/off ratio, retention time, and cycling stability. PCM devices have fast switching speed and high durability. Moreover, this type of device can gradually control the crystallization and amorphous processes of functional layer materials, realizing multistate storage.

FeM‐based devices leverage the spontaneous polarization reversal of ferroelectric materials under an applied electric field.^[^ ^60^ ^]^ This type of device is typically composed of asymmetric electrodes with an ultrathin ferroelectric layer in between (Figure 4d). Chanthbouala et al. successfully fabricated ferroelectric devices with a multiferroic material (bismuth titanate (BiFeO_3_)) as the ferroelectric functional layer.^[^ ^61^ ^]^ It has been proven that the movement of Domain Walls in ferroelectric materials can be regarded as a brand‐new physical phenomenon for generating memristor phenomena. Under an applied electric field, the interfacial barrier between the strontium ruthenate (SrRuO_3_) electrode and the strontium titanate (SrTiO_3_) substrate will undergo continuous and reversible changes due to the movement of ferroelectric domain walls, thereby regulating the device resistance. Ferroelectric polymers, e.g., polyvinylidene fluoride (PVDF) and its copolymer with trifluoroethylene P(VDF–TrFE,^[^ ^62^ ^]^ lead zirconate titanate (PZT),^[^ ^63^ ^]^ Hf_0.5_Zr_0.5_O_2_^[^ ^64^ ^]^ have attracted wide attention in this field. Zhao et al. constructed a ferroelectric synaptic device composed of Au/Hf_0.5_Zr_0.5_O_2_ (HZO)/Si.^[^ ^64^ ^]^ The electric field drove the ferroelectric domains in the HZO film (thickness ≤5 nm) to rotate, changing the height/width of the Schottky barrier at the metal–ferroelectric interface, thereby regulating the tunneling current. The device has an on/off ratio ≈500, and a retention time ≈10^4^ s. Besides, it successfully emulated various biological synaptic functions. Such devices usually have good stability, retention rate, and fast switching characteristics.

### 3.2 Three‐Terminal Synaptic Device

Three‐terminal synaptic devices typically contain source/drain electrodes, a gate, and a channel layer, the gate and channel layer corresponding to the pre‐synaptic and post‐synaptic membranes, respectively. The synaptic weight is independently regulated by the gate, and the information is transmitted through the channel layer. So compared to two‐terminal synaptic devices, three‐terminal synaptic devices exhibit more stable currents and can simultaneously achieve signal modulation, processing, and transmission. However, they are less suitable for large‐scale array integration. In 2010, Lai et al. constructed a synaptic transistor composed of a silica insulating layer (SiO_2_), a poly [2‐methoxy‐5‐(20‐ethylhexoxy) ‐p‐phenylene vinyl] conjugated polymer layer (MEH‐PPV), a rubidium silver tetraiodide (RbAg_4_I_5_) ion‐conducting layer, and an Al/Ti electrode. Under the action of the electric field, Ag^+^ and I^−^ in RbAg_4_I_5_ can diffuse into the MEH‐PPV polymer.^[^ ^65^ ^]^ When the external electric field is removed, the polymer can retain a certain amount of ionic charge. These residual ionic charges can adjust the carrier concentration of the channel through the electrostatic coupling effect, thereby achieving the regulation of the channel's conductivity. A single device can realize excitatory postsynaptic current (EPSC) and STDP. The current three‐terminal synaptic devices are mainly categorized into floating‐gate transistors (FGT), electric‐double‐layer transistors (EDLT), electrochemical transistors (ECT), and ferroelectric field‐effect transistors (FeFET) (Figure 4e–h).

In FGT synaptic devices, the electronic charge can migrate to the floating gate and be captured due to the quantum tunneling effect under the effect of gate voltage regulation.^[^ ^66^ ^]^ The core lies in embedding the regulating gate layer into the dielectric layer (Figure 4e). Due to the tunneling effect, charges can be easily injected into the floating gate under an electrical field, and information storage is achieved because of the existence of the tunneling layer. Hafnium oxide/hafnium disulfide (HfO_x_/HfS_2_)^[^ ^67^ ^]^ can be used as floating gate layers, and MXenes^[^ ^68^ ^]^ and perovskite quantum dots materials^[^ ^69^ ^]^ can be introduced to prolong the charge retention time. To address the issue of limited accuracy caused by uniform charge capture in traditional floating gate devices, Pei et al. realized charge gradient storage through multistep quantum dot floating gates.^[^ ^69^ ^]^ A stepped Al_2_O_3_ dielectric layer was formed on a SiO_2_/Si substrate, and CsPbBr_3_ perovskite quantum dots (QDs) were introduced to form floating gate layers of different thicknesses (5/10/15 nm). The height difference of the steps forms a gradient barrier, realizing selective tunneling and zoned storage of charges under the coupling of optical/electrical signals. Compared with the traditional Si‐float gate, the photogenerated carrier density of this device is increased by 10^3^ times. FGT synaptic devices can precisely control and store charges for a long time, achieving multistate storage.

EDLT synaptic devices mainly rely on ion migration in the gate dielectric layer and channel layer under gate voltage, simulating the synaptic plasticity based on the double‐layer effect at the interface. EDLT synaptic devices usually possess low operating voltages due to the electric‐double‐layer effect.^[^ ^70^, ^71^ ^]^ EGT devices are usually prepared using polymer electrolytes,^[^ ^72^ ^]^ ion liquid,^[^ ^73^ ^]^ ion gels,^[^ ^74^ ^]^ metal oxide semiconductors (e.g., tin oxide),^[^ ^75^ ^]^ and organic semiconductors (e.g., poly(3‐hexylthiophene) (P3HT).^[^ ^76^ ^]^ Based on the high absorption coefficient of P3HT in the deep ultraviolet band (DUV: 200–300 nm), Jiang et al. coated P3HT thin films (with a thickness of ≤100 nm) on polyethylene terephthalate (PET) and constructed a low‐energy consumption flexible synaptic device.^[^ ^76^ ^]^ The device has a low energy consumption (fJ level) under DUV optical stimulation. EDLT synaptic devices have low operating voltage and energy consumption, which is particularly prominent when operating at low frequencies. (Figure 4f).

Synaptic functions in ECT synaptic devices are mainly realized by ion migration in the gate dielectric and the channel with redox properties.^[^ ^77^ ^]^ (Figure 4g) The Channel layer can adopt PEDOT: PSS.^[^ ^78^ ^]^ Gkoupidenis et al. coated a PEDOT: PSS thin film as the functional layer on a glass substrate with an Au electrode to construct an OECT device.^[^ ^78^ ^]^ When a positive pulse is applied to the gate electrode, cations are injected from the electrolyte into the PEDOT: PSS function layer, causing the latter to be de‐doped. After the pulse is removed, the cations return to the electrolyte, and the PEDOT: PSS layer is reversibly doped back to its initial state. Single‐walled carbon nanotubes (SWCNTs) can also be applied in ECT synaptic devices.^[^ ^79^ ^]^ The electrochemical potential difference between single‐walled carbon nanotubes and water/oxygen REDOX pairs can be regulated by applying a gate electric field, thereby controlling the output current. ECT synaptic devices typically have low driving voltage, low‐power consumption, and good biocompatibility.

FeFET synaptic devices achieve modulation of channel carriers through changing the ferroelectric material's polarization state, thereby simulating synaptic plasticity (Figure 4h).^[^ ^80^ ^]^ Ren et al. developed an all‐inorganic flexible ferroelectric field‐effect transistor, which has a switching ratio of 10 and an operating voltage of ±6 V. In addition, after 500 bending cycles under a 4 mm deformation, the device still maintains stable performance.^[^ ^81^ ^]^ Ferroelectric materials are usually adopted as the gate dielectric.^[^ ^82^, ^83^ ^]^ This type of device has a fast operating speed, low power consumption, and rapid erasure and writing (**Table** 1).

**Table 1:** The performance comparison of different types of synaptic devices.

Type	Materials	Operating voltage [V]	On/off ratio	Retention time [s]	Endurance [cycles]	Ref.
ECM	ZnO	6/−6	10^4^	/	80	[43]
ECM	Cu_2_S	0.6/−0.6	10^5^	6 × 10^4^	/	[44]
ECM	Ag/PMMA	20/−10	10^5^	2.5 × 10^3^	400	[45]
VCM	PEDOT/PSS	2/−1.5	/	1.5 × 10^3^	/	[50]
VCM	MoS_2_	3/−1	10^4^	10^4^	10^3^	[54]
PCM	NiO_x_	3/−3	10	10^4^	100	[58]
PCM	SrFeO_x_	2/−2.5	4 × 10^3^	10^4^	10^4^	[59]
FeM	PZT	1.6/−1.3	10^8^	1.7 × 10^5^	100	[63]
FeM	Hf_0.5_Zr_0.5_O_2_	4/−2	10^4^	2.5 × 10^4^	100	[64]
FGT	HfO_x_/HfS_2_	60/−60	10^7^	5 × 10^3^	500	[67]
EDLT	MoS_2_	0.6/−0.6	10^7^	/	/	[74]
EDLT	LaAlO_3_/KTaO_3_/LAO/SrTiO_3_	2.5/−1	10^6^	/	/	[75]
ECT	PEI/PEDOT:PSS	0.5/−0.5	10^4^	10^4^	/	[78]
FeFET	PZT/ZnO	6/−6	1.4 × 10^4^	10^4^	500	[81]
FeFET	P(VDF–TrFE)‐Al_2_O_3_	15/−15	210^5^	10^4^	600	[82]
FeFET	P(VDF–TrFE)/P3HT	10/−10	100	10^4^	100	[83]

### 3.3 Key Materials and Fabrication Technologies

The main part of the synaptic device includes the gate dielectric and the channel layer. The materials of gate dielectric are mainly categorized into polymer electrolytes,^[^ ^84^ ^]^ inorganic solid‐state electrolytes,^[^ ^85^ ^]^ ion liquids, and ion gels.

Polymer electrolytes are usually obtained by dissolving inorganic salts in ion‐coordinated polymers, where oxygen lone pairs in their polymer chains are coordinated with metal cations under the action of an electric field to achieve ion migration. However, their low ion mobilization number limits the high‐speed response performance. Inorganic solid‐state electrolytes have received extensive attention. Their lattice defect channel enables rapid migration of metal cations, but suffers from high interfacial impedance, and it is mainly used in the two‐terminal synaptic device.

Compared with conventional polymer electrolytes, ion liquids and ion gels have high ion conductivity due to the relatively high concentration of constituent ions and weak electrostatic interactions between ions, and are more suitable for use in three‐terminal artificial synapses with high‐speed switching properties.

Ion liquids generally comprise organic cations (e.g., EMIM⁺) and inorganic/organic anions (e.g., TFSI^−^). Cations include quaternary ammonium ions,^[^ ^86^ ^]^ imidazolium ions,^[^ ^87^ ^]^ pyrrolium ions,^[^ ^88^ ^]^ anions include halogen ions,^[^ ^89^ ^]^ and tetrafluoroborate ions.^[^ ^90^ ^]^ Ion gels are usually made of organic polymers and salt electrolyte materials mixed; the polymer molecular chains are connected or entangled with each other to form a spatial mesh structure, and the structural voids are filled with anions and cations as a dispersing medium. They mainly include natural and synthetic polymer gels. Natural polymer gels have the advantages of good biocompatibility and biodegradability, and common materials include chitosan,^[^ ^91^ ^]^ cellulose,^[^ ^92^ ^]^ alginate.^[^ ^93^ ^]^ While synthetic gels have the advantages of high mechanical properties and adjustable mechanical characteristics including polyacrylamide^[^ ^94^ ^]^ and polyacrylic acid.^[^ ^95^ ^]^

Channel layer materials are generally categorized into organic and inorganic semiconductors. Organic semiconductor materials can be divided into small‐molecule compounds and polymers. Small molecule materials (e.g., pentacene, copper phthalocyanine, etc.) are used to prepare high crystallinity films by vacuum evaporation^[^ ^96^ ^]^ and molecular beam epitaxy,^[^ ^97^ ^]^ but the processing temperature is high and the flexibility is poor. Polymeric materials such as P3HT and PEDOT: PSS can be used to prepare large‐area flexible films by solution processing techniques (spin‐coating,^[^ ^98^ ^]^ scrape‐coating^[^ ^99^ ^]^), which are suitable for low‐power flexible synaptic devices despite their low crystallinity. Their low‐dimensional structures (e.g., ultrathin films,^[^ ^100^ ^]^ nanowires^[^ ^101^ ^]^) further enhance the device integration density.

Inorganic semiconductors (e.g., Si, Ge) exhibit high mobility, but most of them are rigid and need high processing temperatures (>800 °C), so it is difficult for inorganic devices based on these materials to be used in flexible electronics.

In recent years, it has been found that some low‐dimensional inorganic semiconductor materials can be used to prepare flexible artificial synaptic devices. 1D metal oxides represented by zinc oxide nanowires^[^ ^102^ ^]^ and tin oxide nanowires^[^ ^25^ ^]^ have lower energy consumption and are more conducive to realizing 3D high‐density integration, which is similar to the crisscrossed nerve fibers in neural‐like systems. 1D metal oxides are usually achieved by solvent synthesis, electrostatic spinning, template‐assisted methods, digital controllable printing methods, etc. 2D layered films represented by graphene,^[^ ^103^ ^]^ graphdiyne,^[^ ^104^ ^]^ and molybdenum disulfide^[^ ^105^, ^106^ ^]^ are also excellent materials for flexible channel layers. They not only have vertical tunneling function and a unique energy band structure, but also can be integrated by large‐area epitaxy. Their quantum‐limited domain effects (e.g., surface effects, size effects) confer excellent electrical properties. They are usually prepared by chemical vapor deposition (CVD)^[^ ^107^ ^]^ and mechanical stripping transfer.^[^ ^108^ ^]^

## 4 Artificial Nervous Systems

### 4.1 Artificial Tactile Nervous System

As one of the five basic senses, touch is the earliest and most fundamental sense developed in the human body, as well as the most widespread and complex perceptual system. Through long‐term research by scholars, significant results have been achieved in the exploration of the tactile nervous system, including the anatomy of skin structure, various types of mechanical receptors, and the processing mechanisms of neural signals, which provide foundations for the study of artificial tactile nervous systems. In this subsection, we first introduce the biological foundation of the tactile nervous system. Second, we introduce bio‐inspired tactile sensors. Finally, we summarize the current research status of existing artificial tactile nervous systems.

#### 4.1.1 Biological Mechanisms of Tactile Sensation

**Figure 5:**
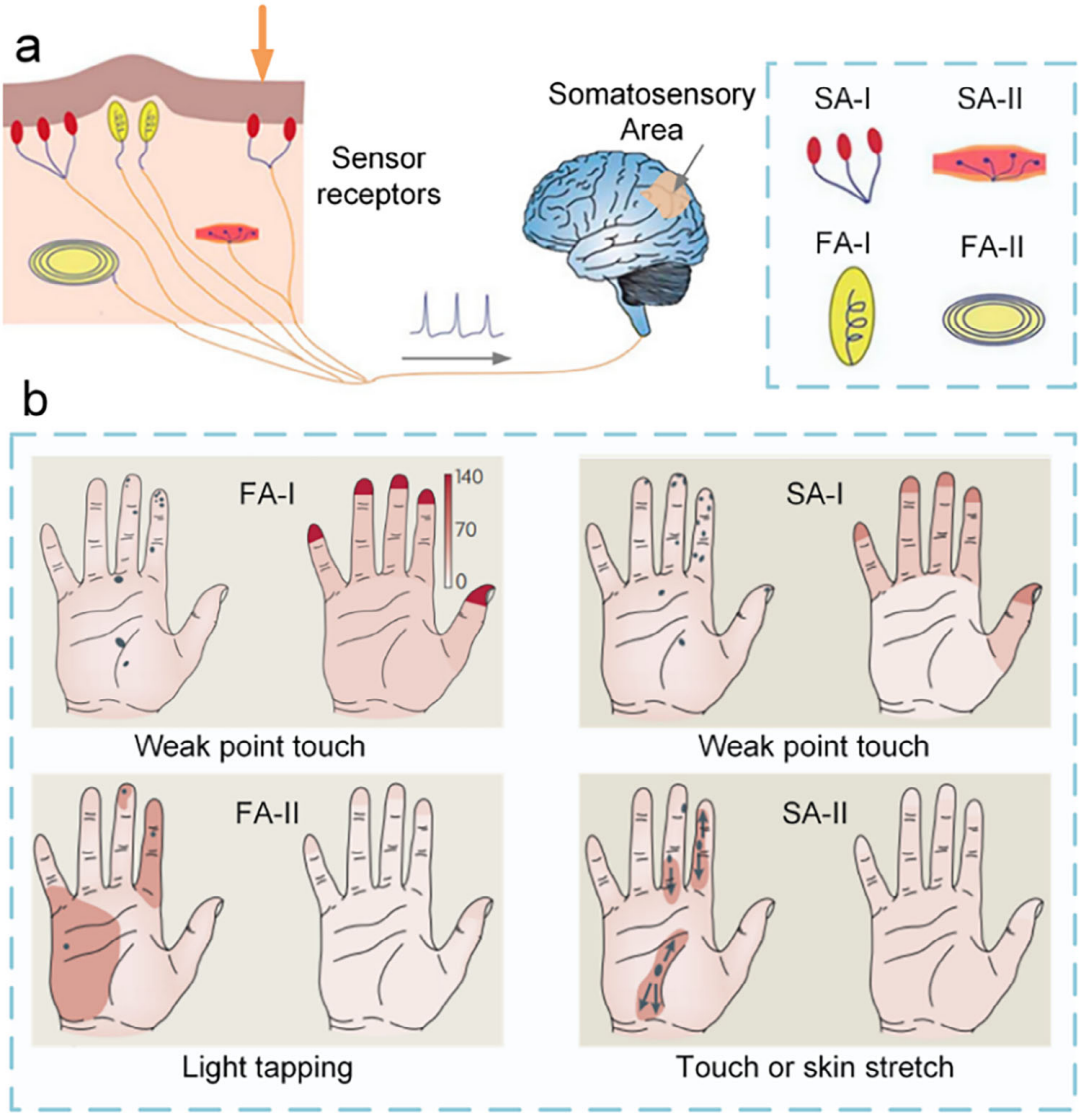
The mechanism of human tactile sensory transmission and distribution of different receptors in the palm. a) Schematic diagram of different parts of the skin's structure and four types of mechanoreceptors, and the process of sensory signal transduction in biological skin. b) Functions and receptive field sizes (left), and density distributions (right) of four types of mechanoreceptors.^[^ ^111^ ^]^ Copyright 2016, Springer Nature Limited.

The skin, serving as the primary interface and protective barrier between the human body and the external environment, plays an important role in facilitating interaction with the surroundings.^[^ ^109^ ^]^ Touch is generally perceived by nerve cells distributed throughout the skin, receiving stimuli such as pressure, vibration, and temperature from the external environment. Among these, receptors play a dominant role; they can convert external stimuli applied to the skin's surface into electrical impulses. Once the primary sensory neurons are activated by the external stimulus information, the receptors process the external information in different coded ways, and the coded information is transmitted along the nerve fibers to the next level of the CNS, thereby forming somatic sensations (**Figure** **5**a).

The receptors present in human skin can be categorized into cold receptors, heat receptors, pain receptors, and four distinct types of mechanoreceptors.^[^ ^110^ ^]^ The four types of mechanoreceptors are Merkel cells, Ruffini corpuscles, Pacinian corpuscles, and Meissner's corpuscles, which are distributed in the dermis of human skin (Figure 5a). They can be differentiated based on their frequency perception range into rapidly adapting (FA) and slowly adapting (SA) receptors, with FA showing sensitivity to relatively high‐frequency dynamic skin deformations, whilst SA are readily elicited by lower‐frequency skin deformations and are able to respond to sustained deformations.^[^ ^111^ ^]^ Merkel cells and Ruffini corpuscles are types of slow‐adapting type I and slow‐adapting type II (SA‐I, SA‐II), and Pacinian corpuscles and Meissner's corpuscles are types of fast‐adapting type I and fast‐adapting type II (FA‐I, FA‐II).^[^ ^112^ ^]^

The distribution and functions of these four types of receptors are different (Figure 5b). FA‐I and SA‐I afferent nerves terminate at the skin surface, with particularly high density at fingertips, where FA‐I afferents outnumber SA‐I afferents. FA‐II and SA‐II afferent nerves terminate in the dermis and subcutaneous fibrous tissue. Specifically, SA‐I receptors were observed in the outermost layer of the skin and are sensitive to static forces and low‐frequency (<5 Hz) dynamic deformations of the skin, making them useful for distinguishing object shapes and high‐resolution textures. Compared to SA‐I, SA‐II receptors are distributed deeper under the skin, typically insensitive to dynamic forces but sensitive to static forces, and can sense tension in the dermis and the collagen fiber chains of subcutaneous tissue, primarily used for measuring skin stretch.^[^ ^113^ ^]^ FA‐I demonstrates sensitivity to skin dynamic deformation at high frequencies (≈5–50 Hz) but is insensitive to static forces, making it suitable for edge contour detection and braille recognition.^[^ ^114^ ^]^ FA‐II is sensitive to high‐frequency vibrations (≈40–400 Hz) but insensitive to static forces, and can be used for detecting transient mechanical events such as rapid slip detection,^[^ ^115^ ^]^ as well as a large number of FA‐II are activated when an object is touched or lowered.^[^ ^116^ ^]^

Furthermore, the pain receptors detect noxious stimuli that result in damage to the body. The human body is endowed with over three million nociceptive receptors, which are distributed throughout the integumentary system, musculature, skeletal system, vascular system, and other visceral organs. These cells are capable of detecting pain caused by mechanical, thermal, or chemical stimuli, including cuts, scrapes, burns, and toxins from insect bites, respectively. These receptors have been shown to trigger intense pain sensations, thereby alerting the organism to move away from potentially harmful stimuli.

Thermoreceptors are used to detect the temperature felt by the skin. It involves cold receptors and heat receptors. Cold receptors begin to sense cold when the skin temperature is below 35 °C. And their stimulation diminishes significantly when the skin surface temperature falls below 5 °C, which is why body parts can become numb and eventually lose sensation when exposed to cold for prolonged periods. Heat receptors start to sense heat when the temperature is above 30 °C, with the strongest stimulation occurring at 45 °C. However, above 45 °C, pain receptors begin to take over, preventing damage to the skin and underlying tissues from excessive heat.

#### 4.1.2 Bioinspired Tactile Sensor

**Figure 6:**
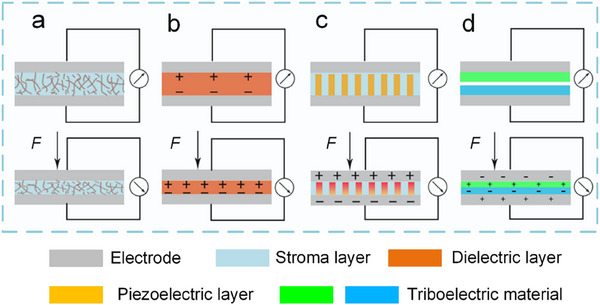
Four types of pressure sensors.^[^ ^117^ ^]^ a) Resistive. b) Capacitive. c) Piezoelectric. d) Triboelectric. Used with permission of [S. Jena, A. Gupta.], from [Review on pressure sensors: A perspective from mechanical to micro–electro–mechanical systems. Sensor Review. 41(3), 320. and 2021 of copyright]; permission conveyed through Copyright Clearance Center, Inc.

By using biological tactile perception as a reference foundation and adopting various design strategies and fabrication techniques, numerous flexible biomimetic sensors have been developed. Flexibility gives the sensor a good contact interface with the functional unit, such as ensuring excellent flexibility when attached to artificial limbs and other controls, and ensuring minimal invasive damage when implantation into biological tissues. According to the working mechanisms, tactile sensors involve resistive, capacitive, piezoelectric, and triboelectric types (**Figure** **6**).^[^ ^117^ ^]^

The working principle of flexible resistive pressure sensors is to measure the resistance's change when they deform under pressure.^[^ ^118^ ^]^ Based on the working mechanisms, they can be further categorized into: the change of material's intrinsic resistivity, the change of resistance at the contact interface, and the quantum tunneling effect in the material composite. The resistive flexible pressure sensors with high sensitivity and simple fabrication processes. However, they also have some drawbacks, such as their stability being greatly affected by temperature and difficulties in signal acquisition.

Enhancing the performance of resistive pressure sensors generally involves considerations in both material selection and microstructural design. The change in resistance of these materials typically results from changes in their resistivity under external pressure. Such materials include carbon nanotubes,^[^ ^119^, ^120^ ^]^ graphene,^[^ ^121^, ^122^ ^]^ liquid metals,^[^ ^123^ ^]^ metal particles,^[^ ^124^ ^]^ and conductive polymers.^[^ ^125^, ^126^ ^]^ When composite materials are used to fabricate pressure sensors, changes in the distance between materials can also lead to changes in resistance. Due to percolation theory and quantum tunneling effects, the combination of conductive materials with low‐conductivity polymers results in the formation of a conductive network, ultimately leading to a reduction in resistance. When subjected to an applied force, resistance decreases with increasing pressure is defined as the negative resistance effect,^[^ ^127^ ^]^ while the opposite is the positive piezoresistive effect.^[^ ^128^ ^]^

The performance of sensors can be significantly improved by designing microstructure arrays, such as spiked arrays,^[^ ^129^ ^]^ spherical arrays,^[^ ^130^ ^]^ columnar arrays,^[^ ^131^ ^]^ and interlocking structures.^[^ ^132^ ^]^ Due to the inhomogeneity of the stress distribution, the top of the pyramidal structure is the most widely used microstructure in applications due to more significant mechanical deformation and sensitivity.

Capacitive flexible pressure sensors have garnered extensive research due to their dynamic responsiveness, stability, durability, high sensitivity, and high resolution.^[^ ^133^ ^]^ Capacitive sensors usually comprise two symmetric electrodes, upper and lower, with a dielectric layer in the center. These share similarities with resistive sensors; the insulating elastic materials and microstructural designs that are used for piezoresistive sensors can be applied as the dielectric layers of capacitive sensors, e.g., Ecological Flexibility (Ecoflex),^[^ ^134^ ^]^ Thermoplastic Urethane (TPU),^[^ ^135^ ^]^ Polydimethylsiloxane (PDMS),^[^ ^136^ ^]^ thermoplastic elastomers.^[^ ^137^ ^]^ Flexible capacitive sensors have the capacity to convert external pressure stimuli into capacitance. As shown in Figure 6b, the distance between the top and bottom electrodes changes under external force, leading to a change in capacitance. The sensing capability of capacitive flexible pressure sensors depends on the softness, dielectric constant, and conductivity of the dielectric layer.

Piezoelectric flexible pressure sensors primarily operate based on the piezoelectric effect. As shown in Figure 6c, pressure causes polarization of the piezoelectric material and generates opposite charges on the upper and lower surfaces, and the size of the pressure can be determined through the collection and analysis of the output electrical signals. Once the applied force is removed, the polarization of the piezoelectric material disappears and returns to the initial state. Various dielectric materials, such as barium titanate,^[^ ^138^ ^]^ PZT,^[^ ^139^ ^]^ polyvinylidene fluoride (PVDF),^[^ ^140^ ^]^ and gel electrolytes^[^ ^141^ ^]^ have been extensively studied. Piezoelectric flexible pressure sensors have the advantage of being self‐powered compared to piezoresistive and capacitive sensors. This type of sensor is suitable for developing self‐powered, low‐power sensing devices. Piezoelectric materials are usually integrated with amplifier elements such as transistors to improve the sensitivity and practical value of pressure sensors.

Triboelectric pressure sensors are based on the principle of triboelectric charging, and the friction nanogenerator (TENG), as a typical representative, which has garnered significant attention. TENG is expected to be a power supply source for wearable electronic devices because of its ability to generate energy efficiently. TENG consists of two types of triboelectric materials with opposite polarity, commonly including nylon, polytetrafluoroethylene (PTFE), and the principal investigator (PI). As shown in Figure 6d, when these materials contact with each other, their surfaces generate electrostatic charges of opposite polarity. After the materials are separated, the electrodes generate opposite charges due to electrostatic induction, creating a potential difference.^[^ ^142^ ^]^

#### 4.1.3 Artificial Tactile Nervous System

After realizing the sensing function and improving the performance of tactile sensors with different types of mechanisms, researchers are committed to constructing a tactile sensing system with “autonomous ideas.”

**Figure 7:**
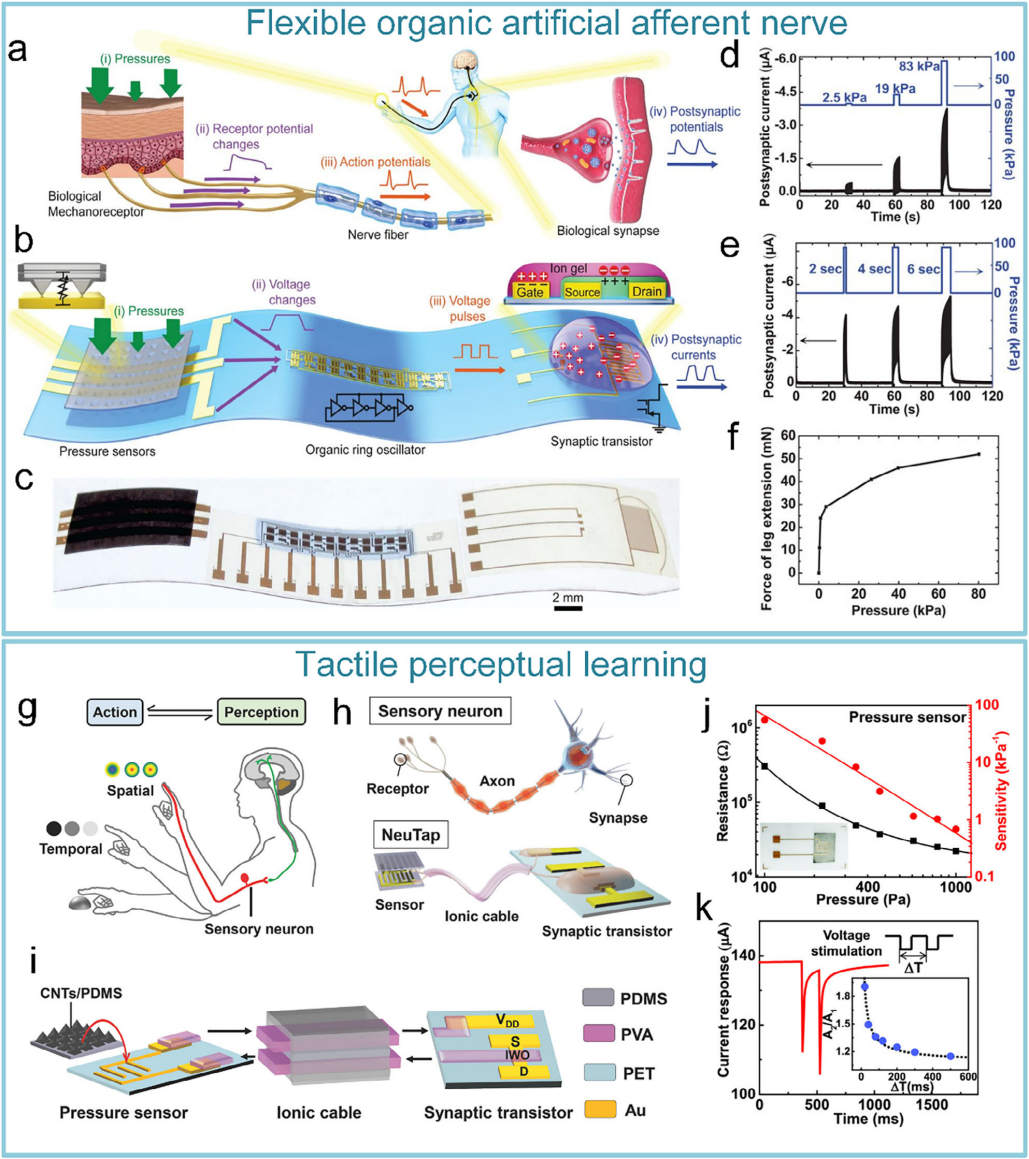
The artificial afferent nerve system comprises pressure sensors and synaptic devices. Pressure sensors convert external pressures of different sizes into voltages of different amplitudes based on the negative piezoresistive effect, which will be coded and processed by a ring oscillator and the artificial synaptic device integrates the spatiotemporal information based on spike‐frequency‐dependent plasticity, and drives the effector by amplifying the postsynaptic current. a) Human afferent nerve that can sense pressure stimuli. b) Artificial afferent nerve's components. c) Image of the artificial tactile nerves. d) Postsynaptic current output under three different pressures (duration: 4 s). e) Postsynaptic current output at three different pressure intensities (pressure: 80 kPa). f) Maximum force of the biological muscle under varying pressures (duration: 0.5 s).^[^ ^14^ ^]^ Copyright 2018, The American Association for the Advancement of Science. Schematic design of an artificial tactile neural system based on piezoresistive sensors, ionic cables, and synaptic devices. g) Illustration of the integration of different spatiotemporal information by tactile sensory neurons in the human tactile sensorimotor circuit. h) Comparison of sensory neurons (top) and the artificial tactile system (bottom). i) Schematic of the unit module of the artificial tactile system. j) Changes of sensor resistance and sensitivity at varying pressures. k) Paired‐pulse facilitation (PPF) response of synaptic devices.^[^ ^143^ ^]^ Copyright 2018 WILEY‐VCH Verlag GmbH & Co. KGaA, Weinheim.

As for the artificial tactile sensory nerves, Kim et al. developed the world's first high‐sensitivity artificial tactile nervous system based on flexible organic electronic devices.^[^ ^14^ ^]^ This system has been used for sensing object movement, recognizing braille, and integrating with insect leg neural pathways to achieve hybrid reflex arcs (**Figure** **7**a–c). The artificial tactile nervous system organically combines piezoresistive sensors, artificial synaptic devices, and biological limbs, in which piezoresistive sensors convert external pressures of different sizes into voltages of different amplitudes based on negative piezoresistive effect, which will be coded and processed by a ring oscillator, and the artificial synaptic device integrates the spatiotemporal information based on spike‐frequency‐dependent plasticity, and driving the cockroach limbs by amplifying the postsynaptic current (Figure 7d–f). Inspired by this work, Wan et al. realized a tactile perception processing system that integrates sensing‐processing‐memory functions through a three‐layer bionic structure. Resistive pressure sensors mimic skin mechanoreceptors (Figure 7g–i). Ion cables simulate axons to realize signal transmission, and synaptic devices replicate synaptic plasticity to achieve information integration and processing (Figure 7j,k). Experiments have confirmed that the system can trigger synaptic dynamic responses based on the frequency differences of mechanical stimuli through the ion/electron coupling interface to form a differentiated memory storage pattern, demonstrating adaptive learning properties of biological neural systems and providing a paradigm for the development of neuromorphic intelligent devices.^[^ ^143^ ^]^

**Figure 8:**
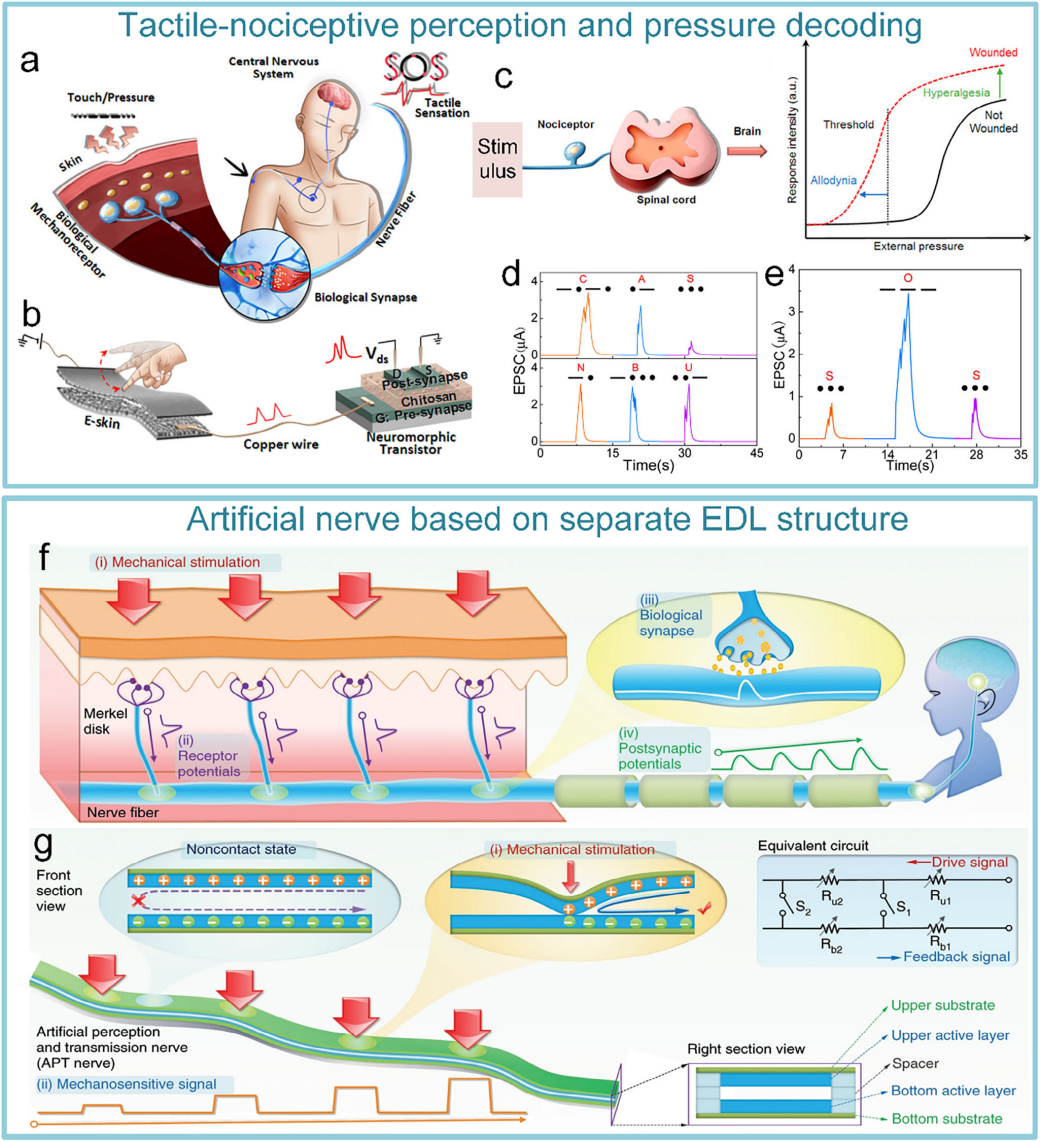
Schematic of an artificial tactile nervous system based on textured piezoresistive sensors and chitosan synaptic devices.a) Tactile afferent nerves. b) Artificial tactile sensory neurons. c) Simulated nociceptive perception function. (d) CAS and NBU Morse code. e) SOS Morse code signals at 7.84 kPa pressure with varying durations (short: 0.1 s, long: 0.5 s).^[^ ^144^ ^]^ Copyright 2020, American Chemical Society. Functional validation diagrams of artificial tactile nervous systems. f) Transduction process of tactile signal. g) Working mechanism of the artificial perception and transmission nerve (APT).^[^ ^145^ ^]^ Copyright 2020, The Author(s).

After realizing the fundamental tactile perception function, the researchers worked to improve the system's performance. Yu et al. realized artificial tactile perception neurons utilizing electronic skin with a neuromorphic device (**Figure** **8**a,b). Wherein, textured micro‐structured PDMS pressure sensors are used to mimic skin mechanoreceptors, exhibiting a sensitivity of ≈2.1 kPa^−1^ and a response time <50 ms. Ion‐electronic chitosan‐gated oxide neuromorphic device, as information‐processing components, exhibited synaptic weight updating (>10⁴ times) and STDP. An artificial tactile sensing system is constructed by integrating textured electronic skin with chitosan‐gated neuromorphic transistors. The system can realize tactile‐nociceptive joint perception and accurately reproduce the four stages of biological nociceptive sensitization (including relaxation, threshold, ectopic nociception, and nociceptive hypersensitivity) (Figure 8c). Furthermore, the system can decode tactile “Morse code” (Figure 8d,e),^[^ ^144^ ^]^ providing a new paradigm for developing intelligent prosthetics with nociceptive warning capabilities. To further improve the system response speed and other performance parameters. Liao et al. developed the bionic tactile sensory nerve with integrated signal perception, transmission, and recognition capabilities (Figure 8f,g). Leveraging the bionic double electric layer microstructure, the graphite film/paper‐based flexible heterojunction is used to realize low‐power signal transmission (static zero‐power consumption). And the system exhibits flexibility, fast response (<21 ms), robustness, and durability (>10⁴ cycles). Furthermore, the spatio‐temporal dynamic logic encoding mechanism is introduced to enable touch sensing artificial nerve mimicking the spatiotemporal dynamic logic of biological neural networks through microcellular conductance gradient modulation. And multifunctional touch interactions were performed for validation, successfully simulating multimodal sensing (pressure/shear force/vibration) of skin mechanoreceptors.^[^ ^145^ ^]^

**Figure 9:**
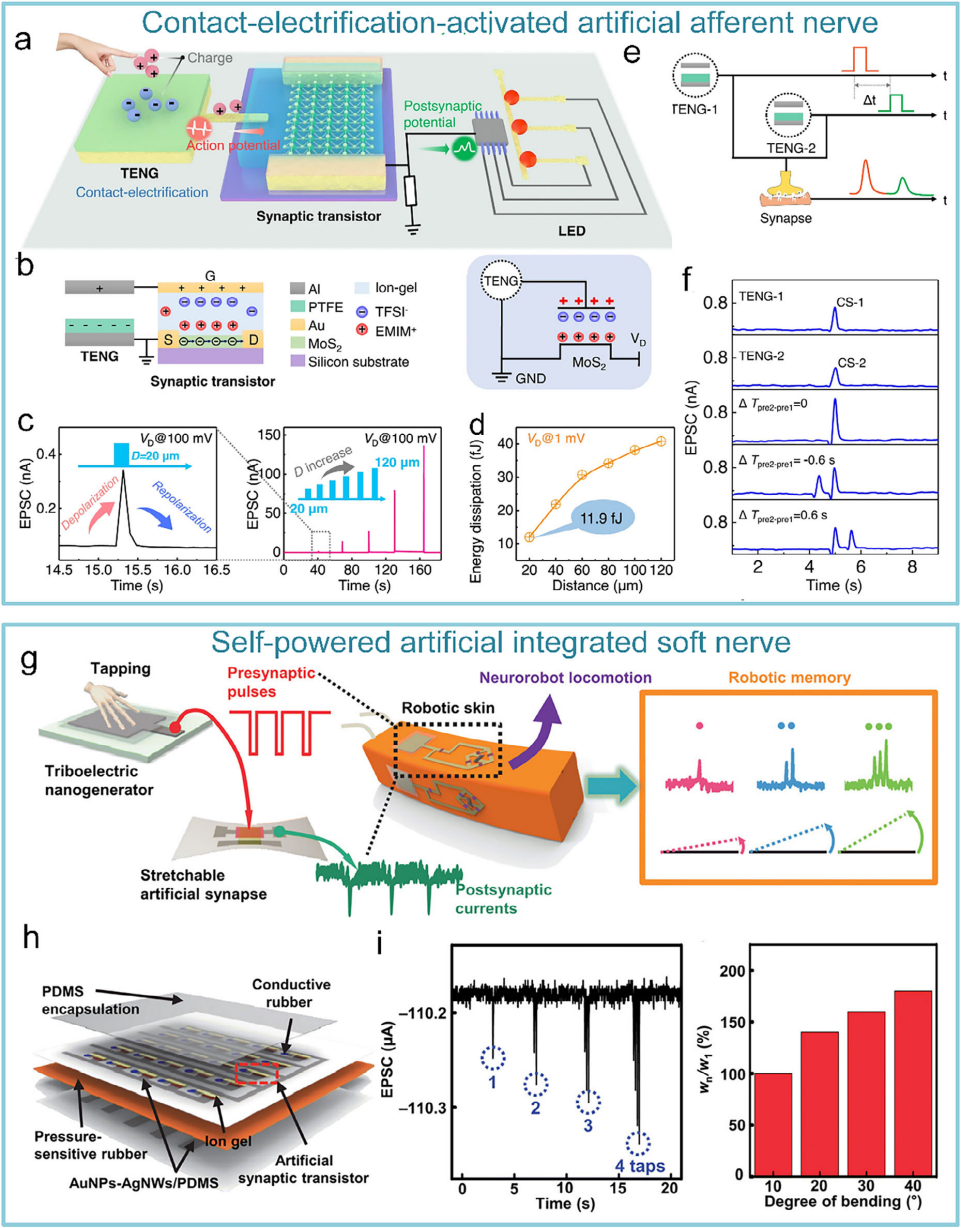
Self‐powered artificial tactile system based on nanogenerators and MoS_2_ artificial synapses. Self‐powered artificial tactile system based on TENG and MoS_2_ artificial synapses. TENG can convert external contact/friction stimuli, such as displacement, pressure, and other tactile stimulation modes, into electrical signals to stimulate synaptic devices. a) Schematic of artificial afferent nerves: including TENG, synaptic transistor, and functional circuit. b) Equivalent modeling diagram of components of the artificial afferent nerve. c) Excitatory postsynaptic currents (EPSC) responses at different distances. d) Ultralow energy consumption at single pulse (11.9 fJ/pulse). e) System equivalent schematic under dual‐TENG modulation. f) EPSC responses at different spatiotemporal stimulations.^[^ ^146^ ^]^ Copyright 2021, The Author(s). g) Signal memory decoding process of soft neuro‐robots. h) Detailed structure of the system. i) EPSC and Bending angle of soft robot after different numbers of taps.^[^ ^147^ ^]^ Copyright 2019, The American Association for the Advancement of Science.

Combining nanogenerators with synaptic devices, self‐powered artificial nervous systems were developed. Yu et al. reported an artificial tactile afferent nerve with tactile‐like sensing and information processing functions (**Figure** **9**a). Converting external contact/friction stimulation (such as displacement, pressure, and other tactile stimulation modes) into electrical signals, realizing passive sensing and self‐powered characteristics (Figure 9b). This energy harvesting approach overcomes the bottleneck of traditional tactile sensors relying on external power sources. Triboelectric signals induced at different distances can activate the ion‐gel‐gated MoS_2_ synaptic device (Figure 9c). Benefiting from the self‐powering feature of the nanogenerator and the ion‐confined transport mechanism of the synaptic device, the system achieves a single‐pulse energy consumption as low as 11.9 fJ, which is three orders of magnitude lower than conventional CMOS synaptic circuits, meeting the low power requirements of distributed neural networks (Figure 9d). Moreover, the artificial tactile afferent nerve exhibits adaptive spatiotemporal recognition/perception capabilities for external stimuli (Figure 9e,f).^[^ ^146^ ^]^ Shim et al. explored a neural integrated control‐based adaptive soft robot, which can control the adaptive motion of the robot by physically tapping the triboelectric nanogenerator device and processing it through a neural synaptic device (Figure 9g). One of the synaptic devices is prepared from an elastic rubbery electronic material, and the specific process is that the mechanoreceptor is externally stimulated and generates a presynaptic impulse, and then the synaptic device generates a postsynaptic potential that ensures that the soft robot is able to perform adaptive motions based on the robot's memories in a programmable manner when physically tapping on the skin (Figure 9h,i), laying the foundation for the development of adaptive robots in complex scenarios such as disaster relief.^[^ ^147^ ^]^

**Figure 10:**
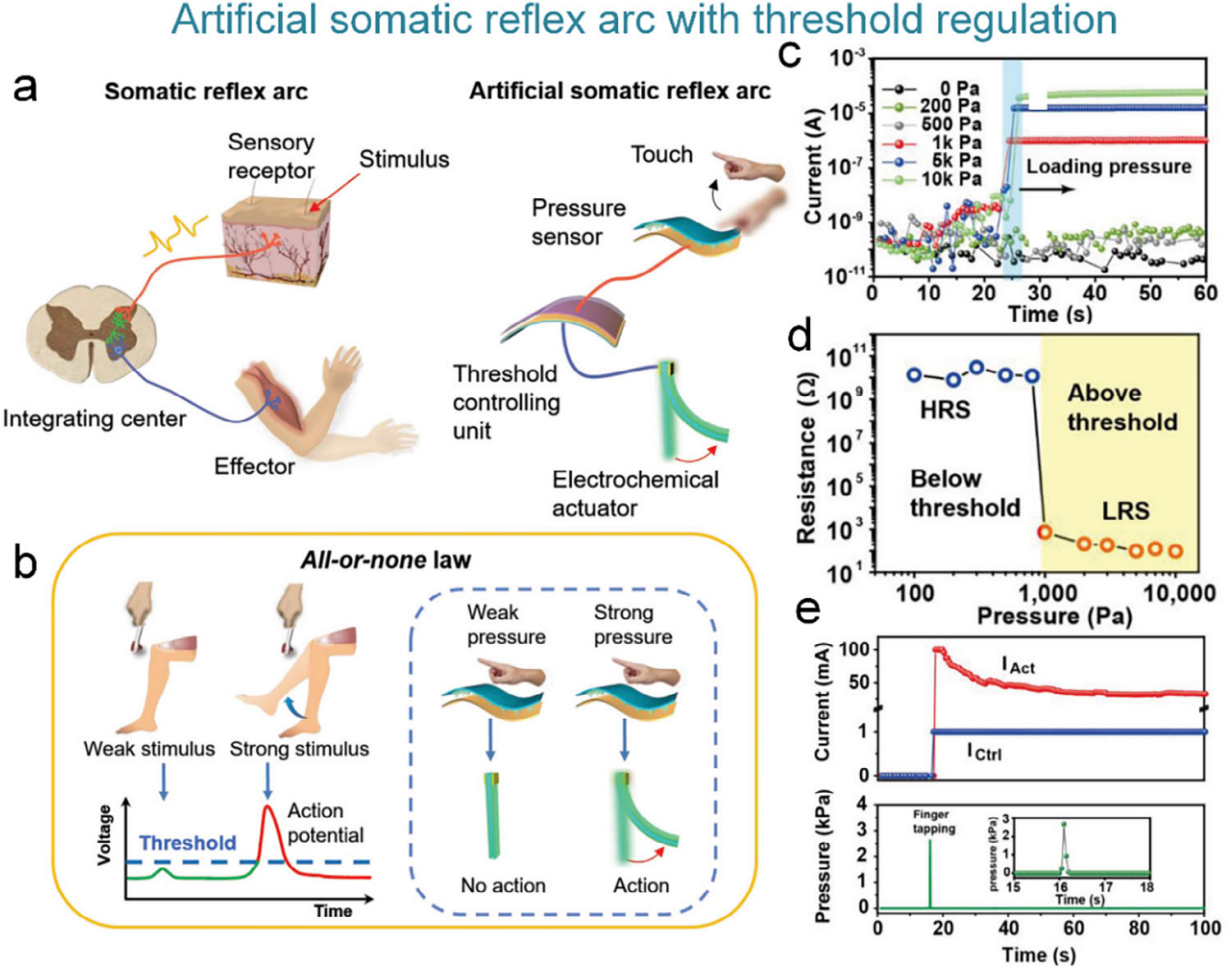
Conceptual illustration of an artificial reflex arc system based on piezoresistive sensors, synaptic devices, and actuators. a) Comparison of the human (left) and artificial somatic reflex arc (right). b) Mimics the knee‐jerk reflex in biological systems, where the reflex is completed when the stimulus exceeds the activation threshold. c) Current response of the system at different pressures. d) Variation of TCU resistance with applied pressure. e) Response currents of the control circuit (Ictrl) and drive circuit (IAct) at above threshold pressure.^[^ ^148^ ^]^ Copyright 2019 WILEY‐VCH Verlag GmbH & Co. KGaA, Weinheim.

Inspired by the structure of biological reflex systems, He et al. constructed an artificial reflex comprising pressure sensors, a threshold control unit (TCU), and an artificial muscle (**Figure** **10**a,b). This system adopts a three‐level architecture of “sensing‐decision‐execution,” flexible pressure sensors mimic skin mechanoreceptors, TCU replicates the signal integration function of the spinal cord, and the artificial muscle corresponds to the biological effector in muscle tissue. By precisely modulating the ion transport threshold of the TCU, the system successfully emulates the “all‐or‐none” response mechanism of the knee‐jerk reflex only when the pressure stimulus exceeded the critical threshold (>1 kPa) (Figure 10c,d). The TCU selectively opened ion channels and triggered the actuator to achieve biomimetic muscle contraction (Figure 10e).^[^ ^148^ ^]^

Lee et al. developed a flexible artificial tactile neuromorphic device with adaptive functions based on the tribo‐capacitive coupling effect. Specifically, a ferroelectric nanocomposite gate dielectric layer was prepared by mixing barium titanate nanoparticles with P(VDF‐TrFE). When tactile stimulation is applied, the dipoles in the ferroelectric gate dielectric will be rearranged due to the triboelectric‐capacitive coupling effect, realizing self‐powered characteristics. Moreover, the tactile memory function was simulated in a 2 × 2 device array. The post‐synaptic current can be modulated by external mechanical stimulation. After the stimulus was removed, the tactile's number and sequence could be identified without additional complex circuits or signal processing procedures. This exploration not only reduces the demand for information processing but also enhances the integration of equipment.^[^ ^149^ ^]^ Wang et al. combined ion‐conducting elastomers with MXene‐based artificial synapses to construct an integrated chip‐type flexible artificial neural tactile nervous system. Specifically, this tactile system is composed of a top electrode layer, an ion‐conducting elastomer layer, an MXene layer, and a bottom electrode layer. Among them, the ion‐conducting elastomer is composed of a porous composite material made of TPU and 1‐ethyl‐3‐methylimidazolium tetrafluoroborate (EMIMBF_4_), which can simulate the ion migration mechanism in the tactile organ and realize the detection of external pressure. In addition, this device can detect extensive joint movements and subtle changes in heart rate.^[^ ^150^ ^]^

### 4.2 Artificial Visual Nervous System

Vision is one of the sensory modalities that plays an instrumental role in the perception and processing of external information by humans. The visual system is the most advanced and functionally sophisticated among all biological sensory nervous systems, capable of efficiently processing optical information, perceiving complex 3D scenes and objects, understanding intricate environments, and recognizing all objects. This section first introduces the biological foundations of the visual nervous system, including the structural components and the corresponding signal transduction processes. Next, the bioinspired artificial vision sensors were briefly reviewed, including the main classifications and corresponding structures. Finally, we summarize the current research status in artificial visual nervous systems.

#### 4.2.1 Biological Mechanisms of Vision

For the human body, more than 80% of information is obtained through the visual system. As the most critical sensory system, it primarily consists of sensory organs (eyes), a part of the CNS (the retina containing photoreceptors and transmission nerves), and the visual cortex of the brain. Notably, one‐third of the cerebral cortex is dedicated to vision.

Most visual perception originates from signal transmission among neurons in the retina. ≈130 million photoreceptors absorb light, and 1.2 million ganglion cells transmit light information from the retina to the cerebral cortex. The specific process is that photoreceptor cells in the retina convert light signals into neural electrical signals, and the dispersed electrical signals are integrated and transmitted to the bipolar cells of the retina. In the process, the horizontal cells regulate photoreceptor activity via negative feedback, ensuring physiological responses remain within an optimal range. Thus, the preprocessing of visual information begins at the bipolar and horizontal cells.

The integrated electrical signals are transmitted to ganglion cells, where amacrine cells filter out redundant visual data. The refined information that has been integrated and processed by various cells is transmitted to the visual center of the brain via the optic nerve. Specifically, following the transmission of visual signals from the eye to the axons of ganglion cells, these signals first reach the optic chiasm, which can be considered a “sorting station” that reorganises signals from both eyes. In this region, left‐hemisphere visual nerves from both eyes merge and project to the left hemisphere of the brain, while right‐hemisphere nerves converge toward the right hemisphere, ensuring the bilateral visual fields are transmitted to the contralateral side of the brain.^[^ ^151^ ^]^

**Figure 11:**
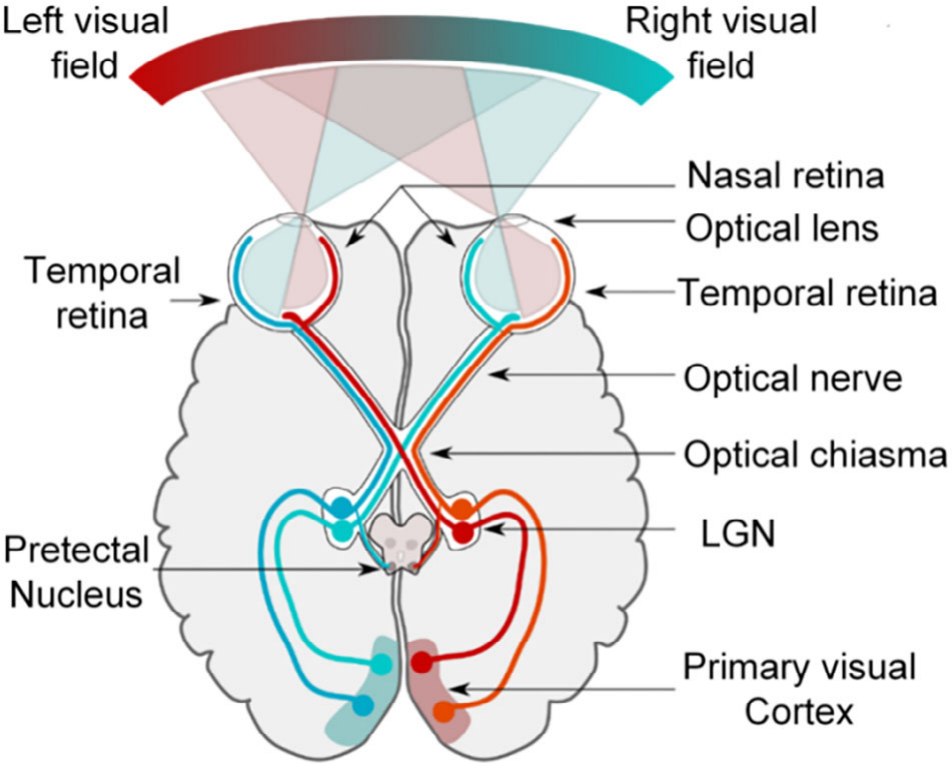
Schematic of human visual pathways.^[^ ^152^ ^]^

After the visual signals are processed away from the optic cross, they are re‐transmitted to three important places: the lateral geniculate nucleus (LGN) in the thalamus, the suprachiasmatic nucleus above the optic chiasma, and the superior colliculus in the corpora quadrigemina. The LGN is responsible for the further processing of visual information, where it is split, twisted, assembled, packaged, and transmitted to the primary visual cortex. The primary visual cortex then performs more detailed feature extraction and classification integration. It not only detects image orientation information, color, motion direction, visual field position, etc., but also distributes the visual information through optic radial nerve fiber bundles to multiple higher brain regions, which carry out subsequent processing in a carefully divided manner. These advanced brain regions further interconnect with more brain regions to influence human behavior and thought (**Figure** **11**).^[^ ^152^ ^]^

#### 4.2.2 Bioinspired Visual Sensor

By taking biological visual perception as a reference basis and adopting different structural designs and processing techniques, researchers have constructed flexible photodetectors (PDs) that can realize optical information perception to simulate visual perception, and realize the correlation between photons and electrons by converting optical signals into electrical signals.^[^ ^153^ ^]^ Current research of PDs primarily focuses on synthesizing novel photoactive materials,^[^ ^154^ ^]^ optimizing device structures,^[^ ^155^ ^]^ exploring new photosensitive mechanisms,^[^ ^156^ ^]^ and proposing different processing techniques.^[^ ^157^ ^]^ PDs have been widely applied in monitoring,^[^ ^158^ ^]^ imaging,^[^ ^159^ ^]^ and communications.^[^ ^160^ ^]^

According to structure and operating principle, current PDs can be categorized into three types: photoconductors, phototransistors, and photodiodes. Photoconductors and phototransistors are horizontal structures, while photodiodes are vertical structures.^[^ ^161^ ^]^

Photoconductors have a simple structure, typically consisting of a semiconductor active layer film and two electrodes. The semiconductor layer acts as a conductive channel, holes and electrons generated in the semiconductor layer under voltage. And it generates more hole–electron pairs due to the photoconductive effect under light, which improves the conductivity of the semiconductor layer. Consequently, when the same external voltage is applied, the photoconductor's photocurrent is greater than the dark current, thereby realizing photodetection.^[^ ^162^ ^]^ Photoconductors exhibit high external quantum efficiency (EQE) and photocurrent. However, their high dark current significantly reduces the detection ratio of the device. Additionally, the horizontal structure usually requires higher operating voltage and longer response time due to its long carrier transport distance.

Similar to photoconductors, phototransistors are horizontally structured devices that comprise three electrodes, a dielectric layer, and a channel layer.^[^ ^163^ ^]^ Phototransistors operate similarly to photoconductors in that they can generate photogenerated carriers and form a photocurrent. As a result, phototransistors with gate voltage modulation have higher switching ratios and EQE compared to photoconductors. However, phototransistors have a relatively reduced response speed and require more complex structures and preparation processes.^[^ ^164^, ^165^ ^]^

Photodiodes differ from the above two structures and are typically vertical structure devices, consisting of an active layer, top and bottom electrodes.^[^ ^166^ ^]^ They operate based on the photovoltaic effect, the energy of photons absorbed by the active layer, and excitons are generated under the light. Electrons and holes after diffusion and transport form a photocurrent. Photodiodes have lower EQE values and response rates compared to photoconductors and phototransistors, but they have higher detection rates due to their low dark current.^[^ ^167^ ^]^

According to the principle and structure of the existing photodetectors, the realization of the light‐sensing function and performance exploration of the photodetectors mainly starts from the three constituent parts of the substrate, electrode, and photoactive layer.

##### Substrates

The substrates of traditional PDs are usually chosen from glass, PMMA, and polyethylene (PE). Flexible and stretchable materials were used to realize flexible bending connections. Among them, flexible materials include PET,^[^ ^168^ ^]^ polyethylene naphthalate (PEN),^[^ ^169^ ^]^ PI,^[^ ^170^ ^]^ stretchable polymer materials involve PDMS,^[^ ^171^ ^]^ carbon cloth,^[^ ^172^ ^]^ paper,^[^ ^173^ ^]^ and biomaterials (hair fibers).^[^ ^174^ ^]^ The devices constructed based on the above substrate materials have excellent flexibility and stable light detection.

##### Electrodes

Current electrode materials focus on conductive polymer materials, metal, and carbon nanomaterials. Metal nanomaterials include copper nanowires,^[^ ^175^ ^]^ gold nanowires,^[^ ^176^ ^]^ and Ag nanowires.^[^ ^177^ ^]^ Carbon nanomaterials include density‐optimized carbon nanotube electrodes,^[^ ^178^ ^]^ graphene electrodes obtained by in situ growth.^[^ ^179^ ^]^ There are also some conducting polymers (e.g., PEDOT: PSS^[^ ^180^ ^]^), conducting oxides (e.g., SrVO_3_^[^ ^181^ ^]^), and novel 2D materials (e.g., MXenes^[^ ^182^ ^]^) employed as electrodes for photodetectors.

##### Active Layers

The light absorption ability of active layer materials is the basis of photodetectors for various functional applications. Inorganic semiconductors have excellent photosensitivity and stability.^[^ ^183^, ^184^ ^]^ Perovskite and organic materials are widely studied due to their high photosensitivity and flexibility. The widely used structure of organic photodiodes is the heterojunction, where the acceptor and donor are comingled.^[^ ^185^ ^]^ The acceptor can choose 6,6‐phenyl‐C61‐butyric acid methyl ester (PCBM), and the donor can choose polymer PBDTT‐ffQx^[^ ^186^ ^]^ or P3HT.^[^ ^187^ ^]^ Perovskite materials are currently commonly used in the field of optoelectronic devices due to their high light absorption. Polycrystalline perovskite films are widely used in various optoelectronic devices due to their excellent optoelectronic properties.^[^ ^188^ ^]^ Self‐powered photodetectors can be realized by combining perovskite active layers with friction electric TENGs composed of PDMS/PET.^[^ ^189^ ^]^ Low‐dimensional perovskite materials have unique crystal structures and properties that provide low defect densities and long photocarrier lifetimes. The main low‐dimensional perovskite materials commonly include QDs,^[^ ^190^ ^]^ perovskite nanoparticles (NPs),^[^ ^191^ ^]^ perovskite nanowires (NWs),^[^ ^192^ ^]^ and perovskite nanosheets.^[^ ^193^ ^]^ Combining the two materials to construct organic/calcite hybrid flexible photodetectors is also a focus in current research.^[^ ^194^, ^195^ ^]^

To mimic visual system that integrates sensing and computational functions, requires different levels of memory for light response signals to achieve complete neuromorphic recognition and memory processing functions. Therefore, researchers are developing neuromorphic devices and systems with light‐sensing and response functions to realize complex biological visual behaviors.

#### 4.2.3 Artificial Visual Nervous System

**Figure 12:**
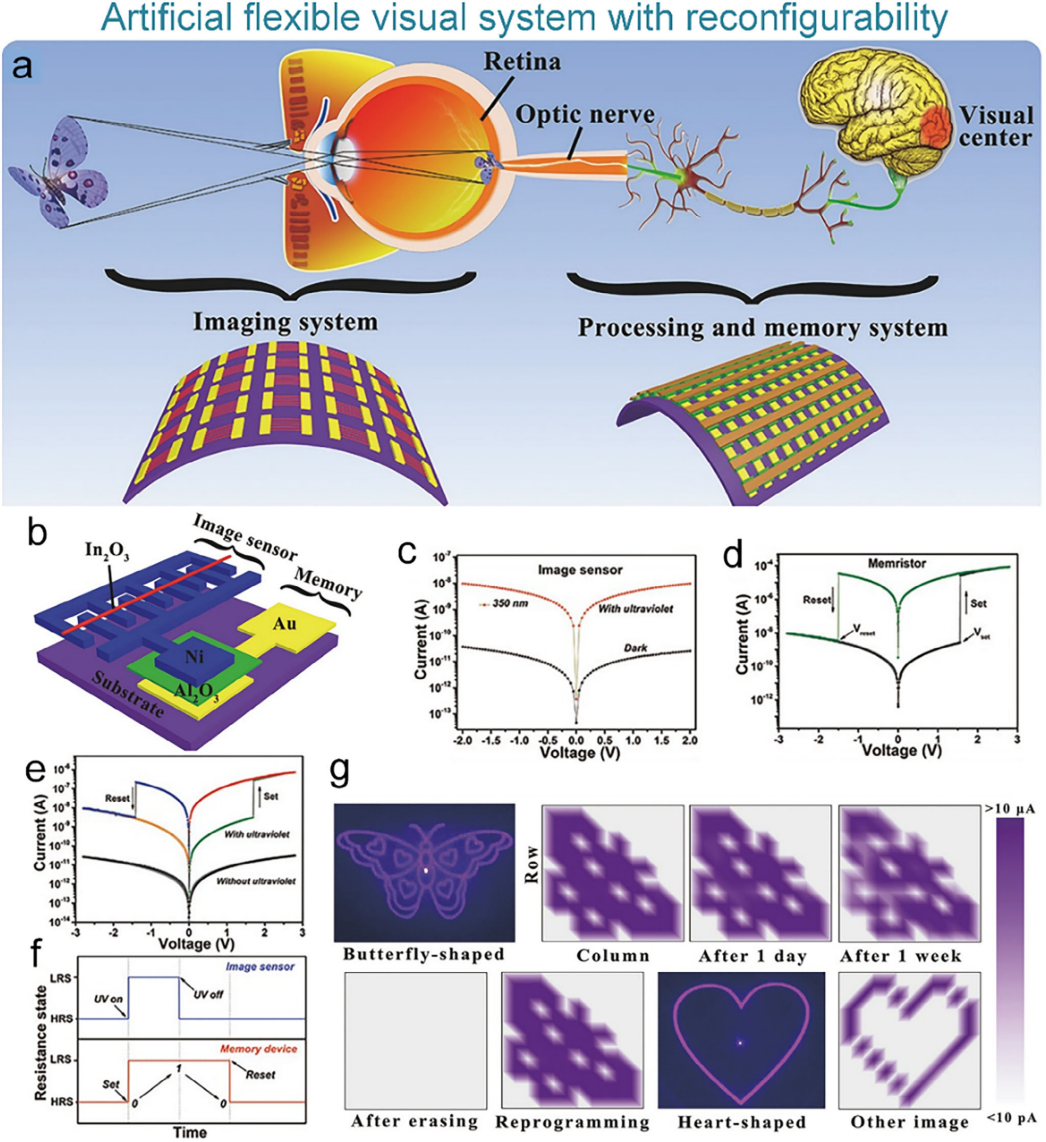
Bio‐inspired visual memory systems comprise UV optical sensors and memristor devices. a) Visual information perception process in the human eyes. b) Equivalent schematic of the bio‐inspired visual memory system. c) I–V curves of the sensor under 350 nm illumination and dark conditions. d) I–V characteristics of the memristor device. e) I–V behavior of the system with/without UV illumination. f) Correlation mechanism between the sensor and memory device. g) Performance evaluation of the system's image‐pattern memorization capability.^[^ ^196^ ^]^ Copyright 2018 WILEY‐VCH Verlag GmbH & Co. KGaA, Weinheim.

To address the issue that the image perception information of artificial visual neuromorphic devices will disappear when external optical stimuli are removed, Chen et al. constructed a bio‐inspired artificial visual perception and storage system. The system integrates an optical sensor array and synaptic device arrays (**Figure** **12**a–d), enabling simultaneous perception and storage of external images. Due to the stored visual information can be erased at negative voltages, the system can be reprogrammed to generate the appropriate image, which demonstrates a strong reconfiguration capability (Figure 12f,g). This innovation offers a novel solution for wearable devices, bionic electronic eyes, and visual impairment assistive technologies.^[^ ^196^ ^]^

**Figure 13:**
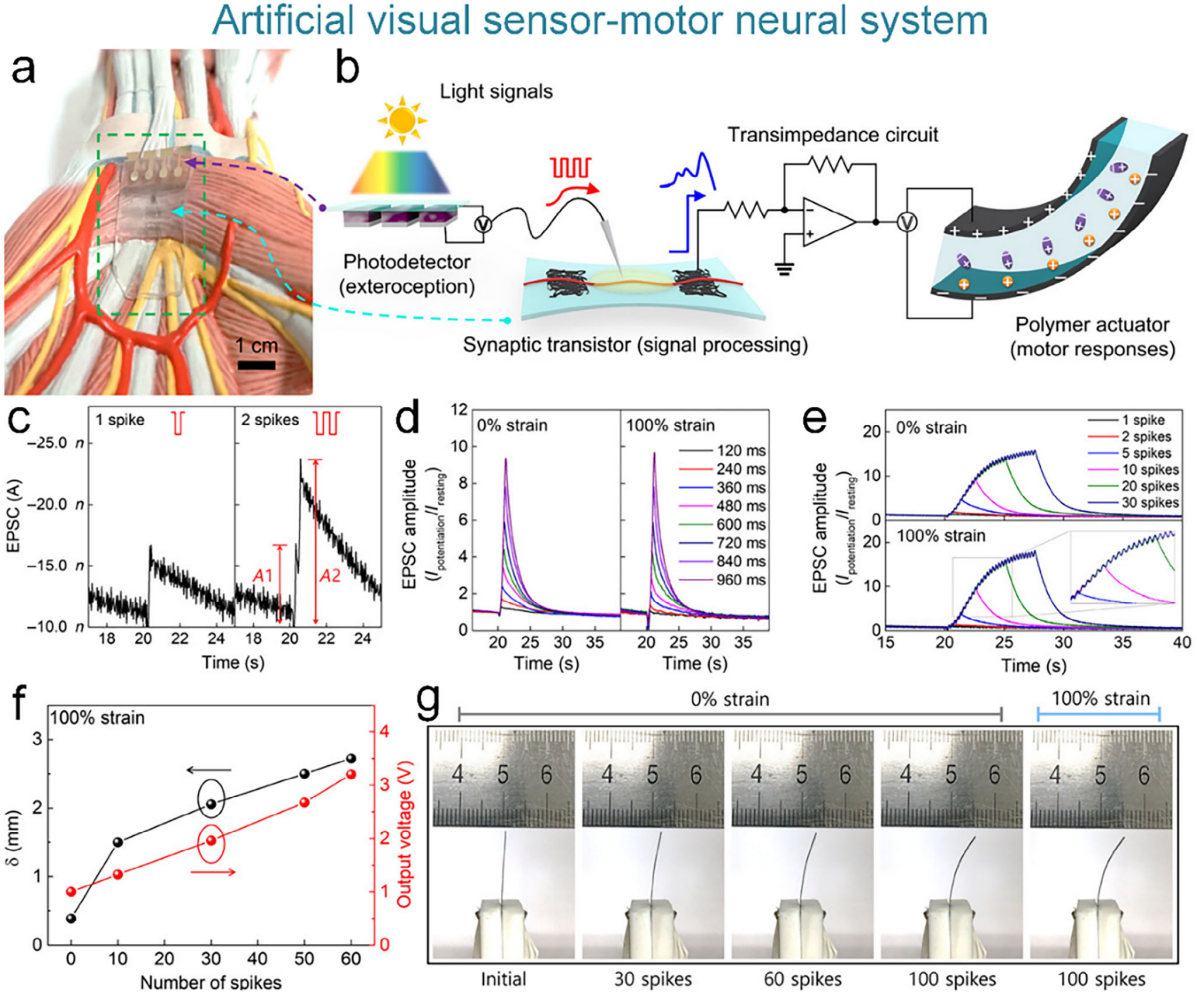
Architecture and performance of the artificial visual perception‐motor neural system.a) Photograph of the optoelectronic synapse. b) Artificial visual system comprising a photodetector, stretchable artificial synaptic device, circuit network, and artificial muscle. c) EPSC and PPF responses of the system under visible light. d, e) SDDP and SNDP responses under 0–100% mechanical strain. f) IPMC actuator's maximum displacement and front‐end device output voltage under 0–60 optical stimulation pulses. g) Deflections of the IPMC under 0–100 pulses.^[^ ^197^ ^]^ Copyright 2018, The American Association for the Advancement of Science.

**Figure 14:**
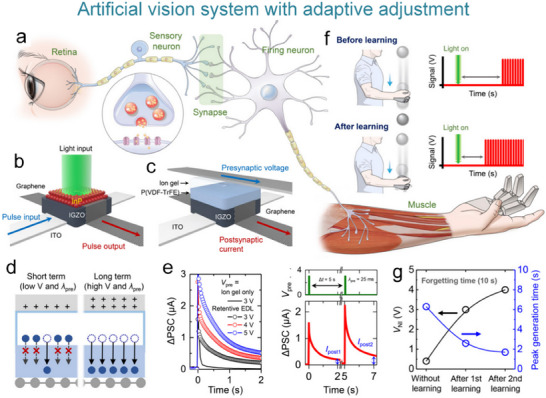
Artificial visual stimulus‐response system based on photodiodes and synaptic devices. a) Schematic of the human visual stimulus‐response pathway. b) Structural schematic of the photodiode. c) Architecture of the artificial synaptic device. d) Operational mechanism of the synaptic device. e) Synaptic response characteristics under varying stimuli. f) Trained learning process in a human subject (ball‐catching task) and corresponding neural signals. g) V_NI_ and peak amplitude plots during different learning phases.^[^ ^198^ ^]^ Copyright 2021, The American Association for the Advancement of Science.

In the field of artificial visual perception neuron systems, Lee et al. constructed an artificial visual sensor‐motor neural system (**Figure** **13**a,b).^[^ ^197^ ^]^ The system performs sensory processing under optical stimulation and triggers contraction reflex responses of the artificial muscle. The photo detector enables optical signal perception. A stretchable synaptic device was fabricated by transferring an organic nanowire onto a 100% pre‐strained SEBS substrate with carbon nanotube‐based source/drain electrodes. This device is capable of executing neuromorphic information processing (Figure 13c–e). Under varying optical pulse stimuli, the actuator exhibits distinct deflection displacements proportional to the number of applied pulses (Figure 13f,g). Kim et al. realized an autonomous artificial visual stimulus‐response system operating under light illumination. The system integrates a quantum dot photodiode, an electrical double‐layer synaptic device, a CMOS‐based artificial neuronal circuit, and a robotic manipulator, corresponding to visual receptors, synapses, neurons, and muscles, respectively (**Figure** **14**a). The photo‐responsive behavior of the artificial visual receptor is achieved through the photoelectric effect of quantum dot films deposited on graphene (Figure 14b). Synaptic plasticity is implemented by controlling ion migration within the electrical double layer (EDL) and modulating the work function of graphene (Figure 14c–e). Signal regulation and transmission are further enabled by the integration of an artificial synapse with a CMOS neuronal circuit. Finally, the integrated system is embedded into a robotic hand, demonstrating artificial reflexive responses to light stimuli (Figure 14f,g). Notably, the pulse‐driven operation reduces power consumption by 40% compared to conventional all‐CMOS circuits.^[^ ^198^ ^]^

**Figure 15:**
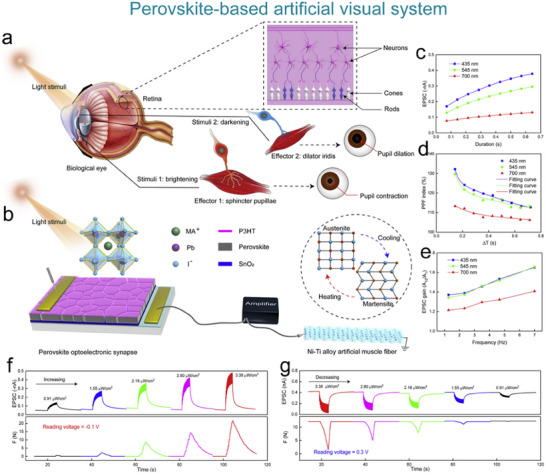
Artificial visual nervous system based on perovskite photoelectric synapses. a) Light‐dependent sensory‐motor processes in the human visual system. b) Artificial visual neural system integrating perovskite synaptic devices, amplification circuits, and artificial muscle actuators. c–e) Relationships between EPSC and pulse duration, paired‐pulse facilitation (PPF) index versus inter‐pulse interval, and EPSC gain versus stimulus frequency under three distinct wavelengths (435, 545, and 700 nm). f) Dynamic variations in EPSC magnitude and corresponding artificial muscle tension under increase and decrease. g) Light intensities demonstrate adaptive bio‐inspired reflex modulation.^[^ ^199^ ^]^ Copyright 2022 Elsevier Inc.

**Figure 16:**
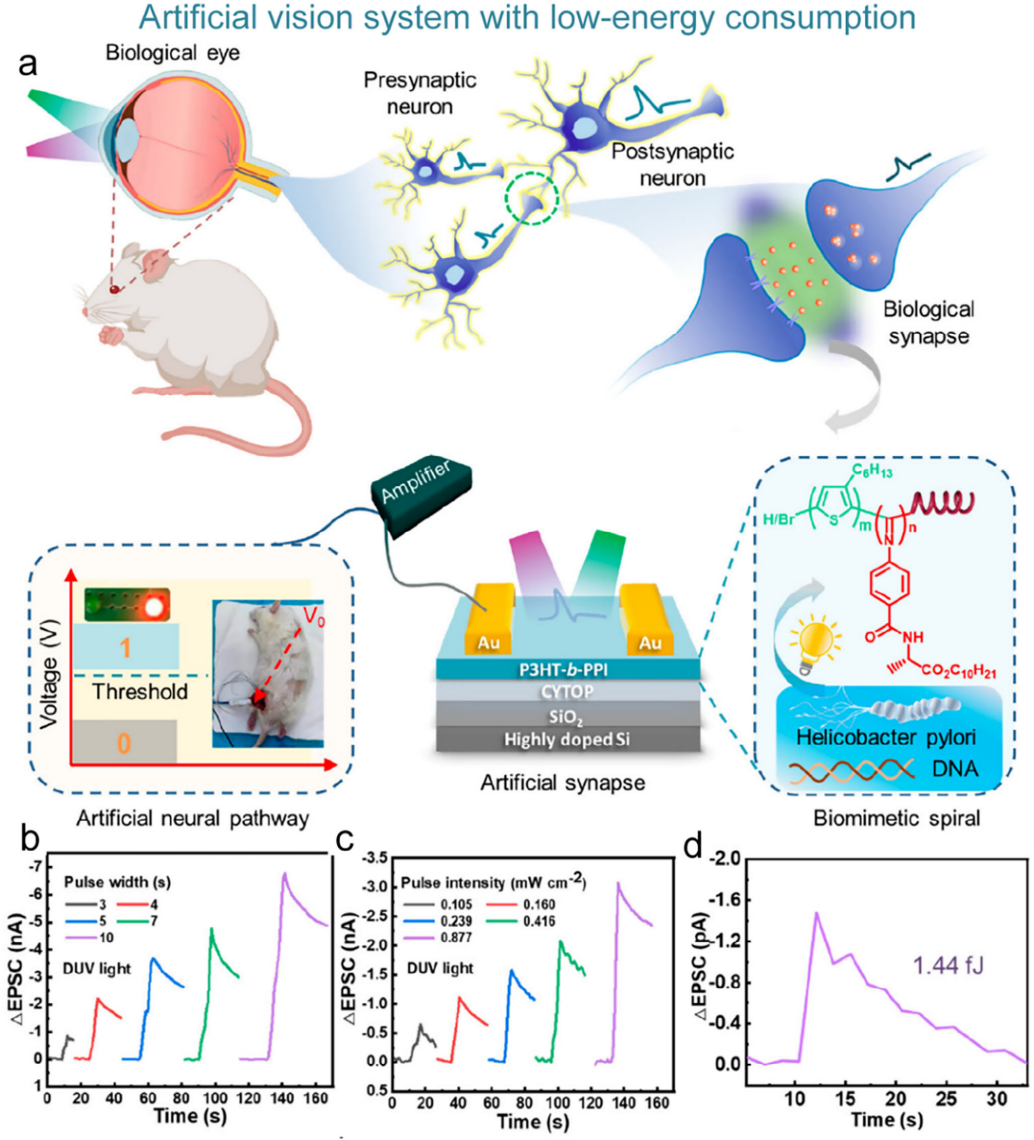
Artificial visual system based on helical‐structured synaptic devices. a) Schematic of biological and artificial visual neural system architectures. b,c) EPSC responses under DUV light with varying pulse widths and intensities. d) Ultralow energy consumption of the device.^[^ ^200^ ^]^ Copyright 2023, American Chemical Society.

Gong et al. developed a perovskite‐based artificial synaptic visual system capable of light‐information perception and processing (**Figure** **15**a,b). They first fabricated a perovskite optoelectronic synaptic device that simultaneously achieves excitatory and inhibitory synaptic functionalities, exhibiting distinct responses to light stimuli at multiple wavelengths (435/545/700 nm; Figure 15c–e). Furthermore, an artificial pupil reflex arc system was constructed, which integrates light signal perception, processing, and actuation to successfully mimic muscle‐controlled adaptive pupil reflexes (Figure 15f,g). This innovation demonstrates the potential of perovskite synapses to emulate biological sensory‐motor coordination.^[^ ^199^ ^]^ Biological perception of visual information is a complex physiological process involving diverse neurons and synapses. Inspired by functional helical structures in nature, Jiang et al. constructed a comprehensive visual neural system with integrated light perception, actuation, and early warning capabilities.^[^ ^200^ ^]^ They synthesized a semiconductor block copolymer featuring a novel helical architecture and employed helical nanowires as the active layer in a synaptic device. The active layer of the optoelectronic synaptic device captures optical signals and converts them into postsynaptic currents, which are then transmitted via amplification circuits to logic circuits and murine leg muscles. This system mimics biological reflex behaviors under varying light stimuli, enabling identification and early warning of harmful optical signals (**Figure** **16**a). The synaptic device exhibits robust long‐term memory effects under DUV light with varying pulse widths and intensities (Figure 16b,c), while achieving energy consumption of 1.44 fJ per synaptic event (Figure 16d).^[^ ^200^ ^]^

### 4.3 Artificial Auditory Nervous System

The functions of the human auditory nerve principally include sound addressing and location, language discrimination and recognition, auditory memory and learning, and hearing protection in the event of noise damage.

#### 4.3.1 Biological Mechanisms of Auditory

**Figure 17:**
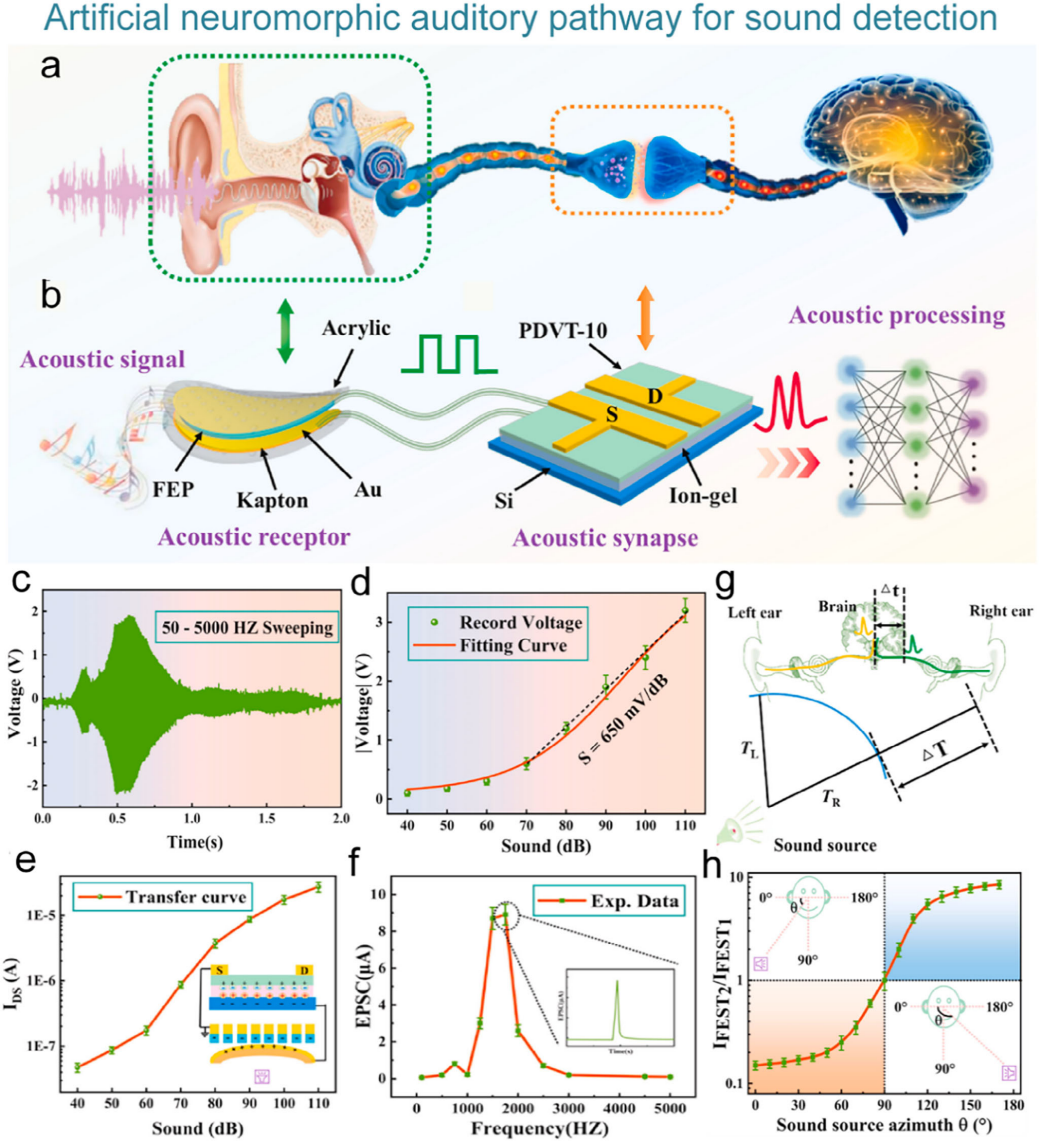
Artificial TENG‐synaptic auditory system and functional validation. a) Schematic of human auditory neural pathways. b) Architecture of the biomimetic auditory system. c) Voltage output spectrum of TENG across 50–5000 Hz frequency range. d) Acoustic intensity‐voltage correlation of TENG. e) Transfer characteristics of the synaptic device under TENG‐driven operation. f) EPSC amplitude modulation with frequency stimulation. g) Experimental setup for binaural sound source localization. h) Synaptic current amplitude ratio versus sound source azimuth (0°–180°).^[^ ^206^ ^]^ Copyright 2020 Elsevier Ltd. All rights reserved.

Auditory perception fundamentally involves the mechanoelectrical transduction process, where sound wave energy is converted into bioelectrical signals. Specifically, external sound waves vibrate the tympanic membrane in the ear canal, which drives the ossicular chain to transmit vibrations to the oval window membrane. This motion creates fluid waves in the inner ear lymph, inducing potential changes in basilar membrane cells. These electrical signals are relayed via the auditory nerve to the brain's auditory centers for neural integration, ultimately generating auditory perception. The auditory system consists of three components: the outer ear (comprising the pinna and ear canal for sound transmission to the middle ear), the middle ear (facilitating efficient energy transfer from air to inner ear fluids), and the inner ear, where hair cell receptors convert mechanical sound energy into receptor currents. These signals are subsequently transmitted to the CNS for further processing (**Figure** **17**a).^[^ ^201^, ^202^ ^]^

#### 4.3.2 Bioinspired Auditory Sensor

Traditional approaches of auditory signal processing employ continuous Nyquist‐rate sampling for application‐specific frequency ranges to capture auditory inputs. These analog signals undergo analog‐to‐digital conversion followed by digital processing to generate auditory frames. However, this signal transformation paradigm exhibits substantial power consumption and inherent risks of critical information loss during quantization.

Therefore, exploring novel neuromorphic electronic devices capable of simulating the functions of the auditory system will contribute to a great breakthrough in artificial neuroauditory systems. Such advancements hold transformative potential for cochlear implants, speech recognition technologies, and robotic auditory perception. Recent advances in bio‐inspired auditory sensing include: the novel auditory sensor inspired by spider webs,^[^ ^203^ ^]^ the binaural neuromorphic auditory sensor with programmable gate arrays,^[^ ^204^ ^]^ and the flexible trapezoidal piezoelectric membrane with multiple electrode channels.^[^ ^205^ ^]^ Furthermore, neuromorphic systems combining auditory perception with motor reflex circuits have been successfully implemented, demonstrating closed‐loop sensory‐motor coordination capabilities.

#### 4.3.3 Artificial Auditory Nervous System

Inspired by the acoustic transduction mechanism of humans, Liu et al. developed an artificial neuromorphic auditory pathway for sound detection,^[^ ^206^ ^]^ comprising a triboelectric TENG and an artificial synaptic device (Figure 17b). The acoustic receptor was biomimetically implemented using a flexible TENG, where femtosecond laser processing enhanced its operational bandwidth and sensitivity (129 mV dB^−1^). TENG simultaneously functions as a self‐powering unit for the system (Figure 17c,d). Signal processing was achieved through an electrolyte‐gated synaptic device emulating auditory synaptic plasticity (Figure 17e,f). The auditory neural system integrates dual artificial acoustic receptors and synaptic devices, which can implement sound localization via interaural time difference detection (Figure 17g). The postsynaptic current gradually increased as sound source angles varied from 0° to 180° (Figure 17h), confirming the system's spatial hearing capability. This adaptive neuromorphic architecture with noise‐regulating behavior demonstrates potential in localization resolution compared to conventional threshold‐based systems.

**Figure 18:**
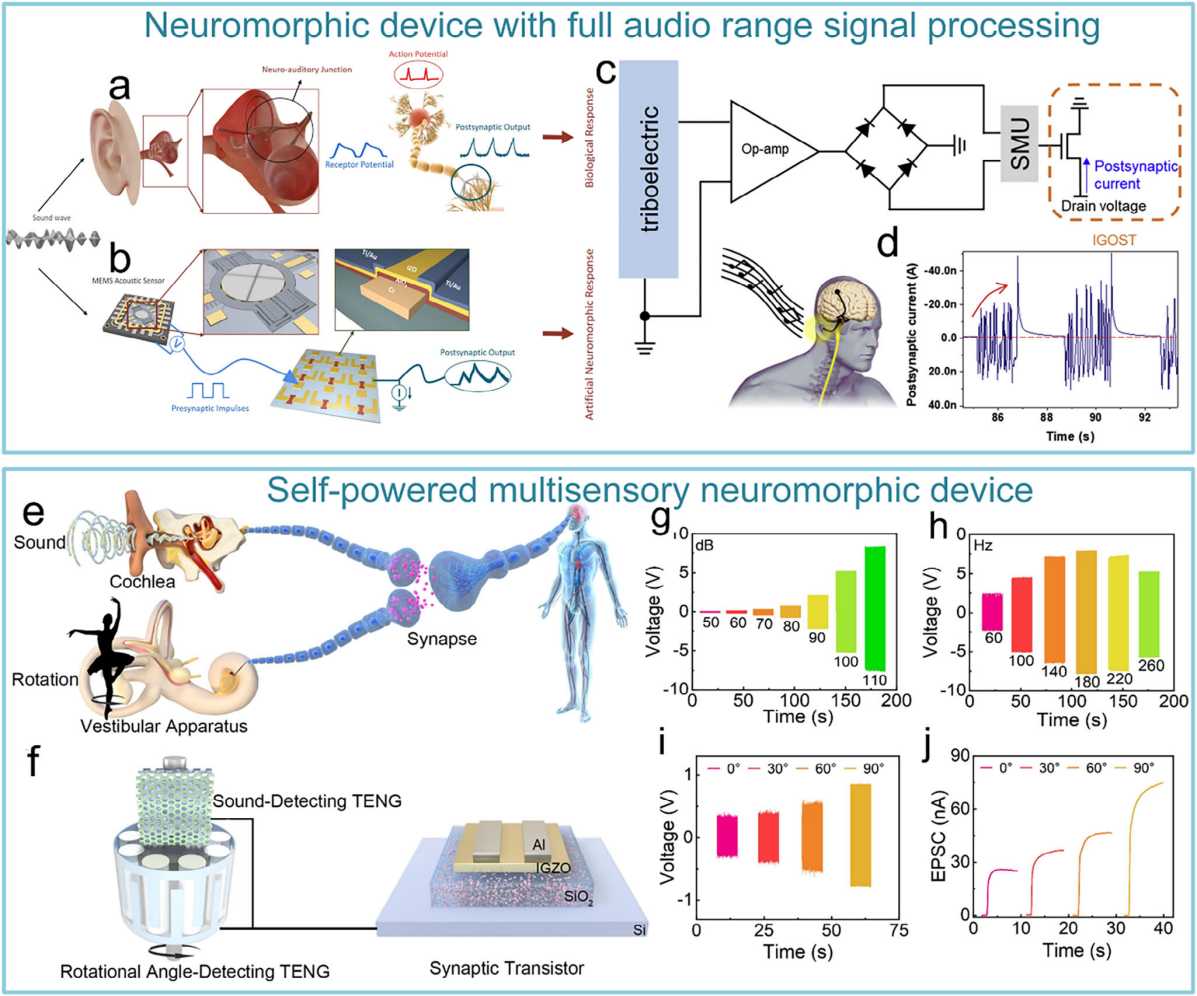
Artificial synaptic device‐based auditory systems. a) Schematic illustrating action potential generation in the human auditory pathway under acoustic stimulation. b) Biomimetic neuromorphic auditory system integrating an energy conversion module (triboelectric/piezoelectric transducer) and synaptic plasticity emulation unit.^[^ ^207^ ^]^ Copyright 2020, The Author(s). c) Circuit architecture of the artificial auditory neural network. d) Synaptically evoked postsynaptic currents under 5 Hz square‐wave acoustic stimulation.^[^ ^208^ ^]^ Copyright 2019 Elsevier Ltd. All rights reserved. e) Biological integration mechanism of auditory‐spatial perception in human neurophysiology. f) Engineered bionic device replicating cochlear–vestibular multimodal integration. g) TENG output voltage versus sound pressure level at 180 Hz excitation. h) Frequency‐dependent TENG response under 110 dB acoustic input. i) Angular sensitivity characterization of TENG‐based acoustic localization. j) EPSC modulation correlated with sound source azimuth.^[^ ^209^ ^]^ Copyright 2025 Elsevier B.V. All rights are reserved.

Bolat et al. developed a neuromorphic auditory system that employs MEMS transducers to convert acoustic signals into electrical outputs, then these electrical signals are amplified and encoded into frequency‐modulated square waves to drive an ion‐gated synaptic device. This system achieves synaptic plasticity responses across the full audio spectrum (20 Hz–20 kHz). Its broadband neuromorphic response provides a hardware foundation for adaptive acoustic processing in intelligent sensing environments (**Figure** **18**a,b).^[^ ^207^ ^]^ Seo et al. developed an artificial auditory neural system that integrates a triboelectric acoustic sensor with an organic electrochemical synapse. Under 5 Hz acoustic stimulation, the system generates triboelectric signals, then these signals are amplified and rectified to trigger a synaptic device, offering an energy‐efficient solution for real‐time acoustic pattern classification (Figure 18c,d).^[^ ^208^ ^]^ Inspired by the multimodal integration of the vestibular and auditory senses, Wang et al. proposed a self‐powered neuromorphic auditory perception system (Figure 18e,f). This system combines a cochlea‐mimetic electrospun micro‐pyramidal TENG with sound pressure response (20–120 dB) and frequency discrimination (50–8000 Hz), paired with a porous SiO_2_‐gated protonic synaptic device, which has excellent proton mobility and energy consumption as low as 4.8 fJ  pulse^−1^ (Figure 18g,h). Utilizing a spatiotemporal fusion algorithm for binaural intensity difference and time difference co‐processing, the system successfully realizes the real‐time sensing ability of small errors in sound source positioning (Figure 18i,j), establishing a hardware framework for adaptive robotic perception in dynamic acoustic environments.^[^ ^209^ ^]^

### 4.4 Artificial Gustatory Nervous System

The gustatory system enables the detection and memorization of chemical taste stimuli while triggering emergency responses upon ingestion of harmful substances. These functions facilitate the intake of appropriate nutrient types and quantities, playing a vital role in maintaining human health.

#### 4.4.1 Biological Mechanisms of Taste

Human taste perception primarily encompasses bitter, sour, salty, sweet, and umami. Each taste modality is detected by taste buds, which are composed of 40–150 taste receptor cells (TRCs) classified into four subtypes: Type I, Type II (including t1r and t2r families), Type III, and Type IV.^[^ ^210^ ^]^ Ion channels, critical components of TRCs, are central to taste transduction. Type II TRCs mediate bitter,^[^ ^211^ ^]^ sweet,^[^ ^212^ ^]^ and umami^[^ ^213^ ^]^ perceptions through molecular mechanisms involving calcium homeostasis modulator 1 (CALHM1)^[^ ^214^ ^]^ and transient receptor potential melastatin 5 (TRPM5) channels.^[^ ^215^ ^]^ Type III TRCs detect sour stimuli via Otop1,^[^ ^216^ ^]^ acid‐sensing ion channels (ASICs),^[^ ^217^ ^]^ and transient receptor potential vanilloid 4 (TRPV4) channels.^[^ ^218^ ^]^ Salty taste transduction is associated with epithelial sodium channels (ENaCs)^[^ ^219^ ^]^ and TRPV1 channels.^[^ ^220^ ^]^

**Figure 19:**
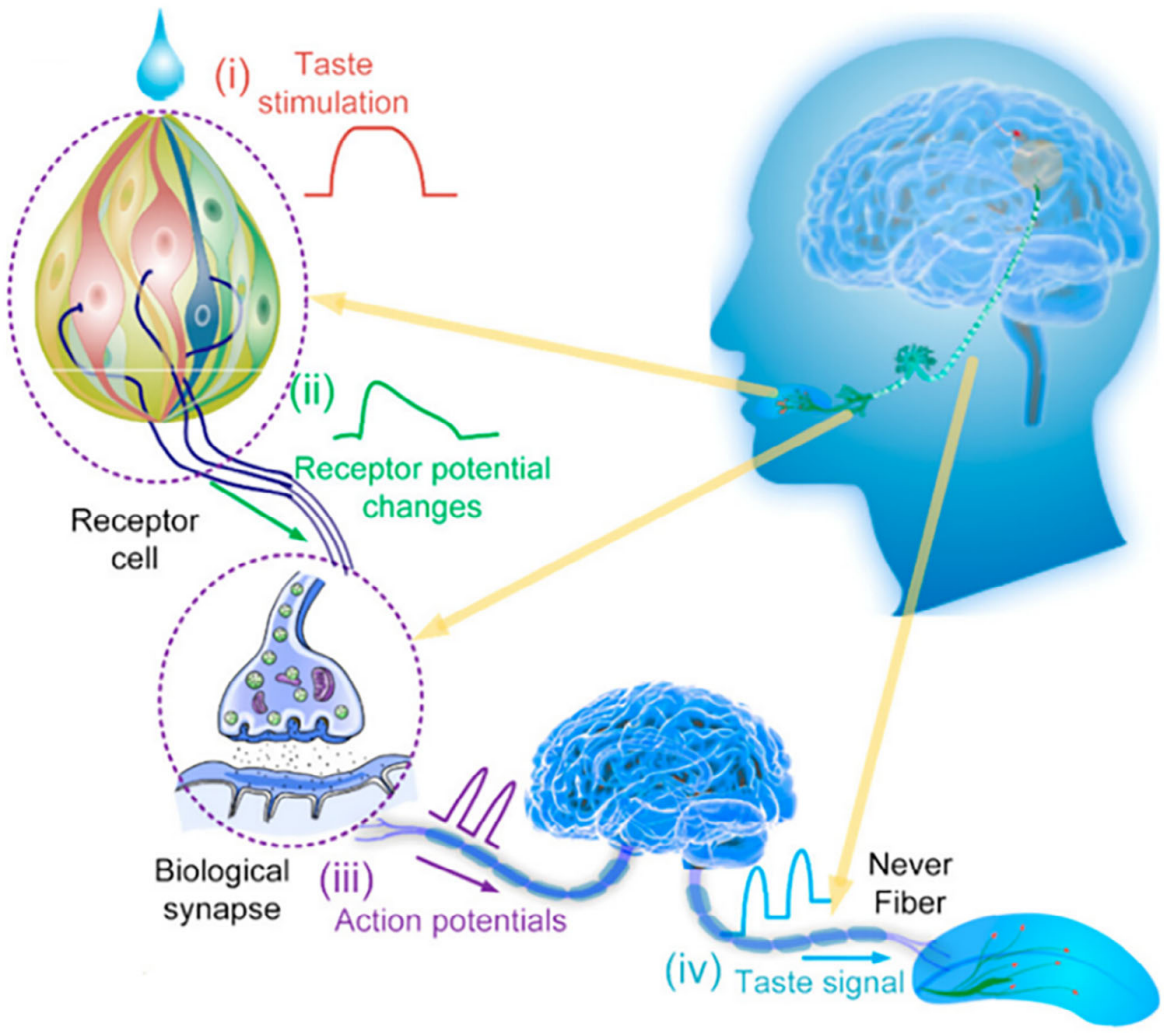
Biological taste signal transduction process.^[^ ^221^ ^]^ Copyright 2023, American Chemical Society.

External tastants activate region‐specific ion channels in TRCs, generating action potentials. These electrical signals are encoded during transmission through nerve fibers to brain regions, ultimately producing taste perception in the CNS and triggering downstream reflex responses (**Figure** **19**).^[^ ^221^ ^]^

#### 4.4.2 Bioinspired Taste Sensor

Inspired by the operational principles of biological gustatory systems, electronic tongues (e‐tongues) have been successfully developed for detecting taste categories and concentrations.^[^ ^222^ ^]^ These devices are categorized into lipid/polymer membrane‐based,^[^ ^223^ ^]^ human taste receptor‐incorporated,^[^ ^224^ ^]^ and voltammetric e‐tongues.^[^ ^225^ ^]^ However, conventional implementations often require complex circuitry for multivariate analysis. Consequently, neuromorphic devices integrating memory and computing architectures are being extensively investigated to construct next‐generation taste‐sensing systems. For instance, bioelectronic tongues utilizing graphene field‐effect transistors have demonstrated capabilities in detecting and distinguishing umami and sweet tastes.^[^ ^226^ ^]^

#### 4.4.3 Artificial Gustatory Nervous System

**Figure 20:**
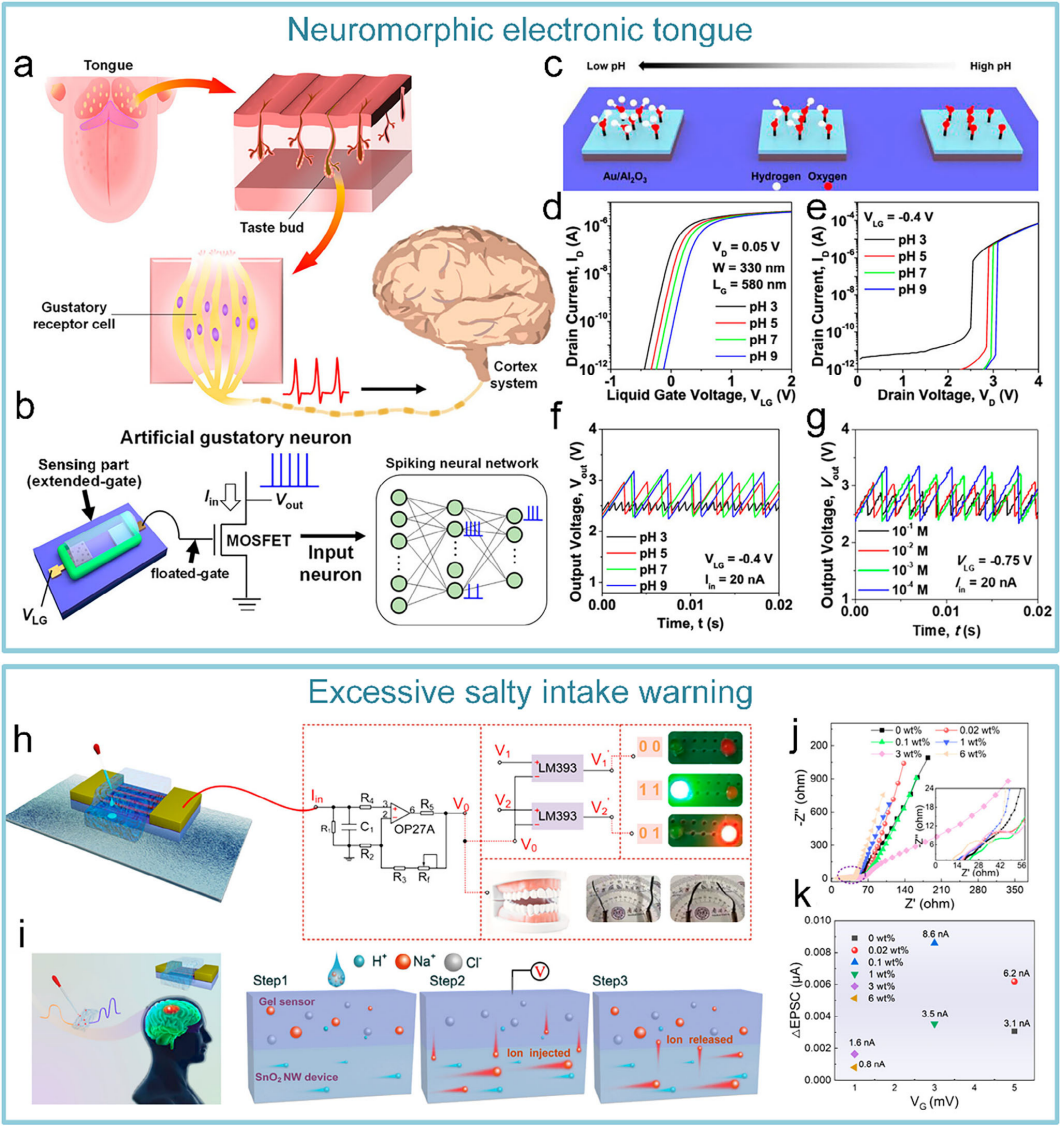
Artificial synapse‐based gustatory system and functional investigations. a) Schematic of signal transduction pathways in the human gustatory system. b) Biohybrid taste system. c) Hydrogen ion‐responsive mechanism in the acid‐sensing unit. d,e) pH‐dependent threshold voltage modulation characteristics. f) Spike frequency modulation under varying pH conditions. g) Sodium concentration‐dependent spiking patterns in the artificial taste‐sensing neuron.^[^ ^227^ ^]^ Copyright 2022, American Chemical Society. h) Intelligent gustatory system with perceptual reflex capabilities for health monitoring and excess intake alert. i) Ion transport‐mediated entrapment mechanism in the artificial taste transduction pathway. j) Electrochemical impedance spectroscopy (EIS) spectra under different saline concentrations. k) Minimum activation voltage and corresponding EPSC across salt concentration gradients.^[^ ^221^ ^]^ Copyright 2023, American Chemical Society.

Han et al. constructed an artificial gustatory neuron composed of chemical sensors and a floating‐gate synaptic device, enabling acid/salt discrimination (**Figure** **20**a,b). This system classifies acidic and salty solutions through chemoelectrical transduction. Chemical sensors detect analyte concentrations, with a floating‐gate synaptic device encoding stimuli into spike trains for processing via spiking neural networks (SNNs). This neuromorphic approach reduces hardware complexity and energy consumption compared to conventional e‐tongues. Acid sensing is achieved through Al_2_O_3_‐functionalized electrodes, where protonation of surface ─OH groups at lower pH increases positive surface charge (Figure 20c). This shifts the transistor's threshold voltage (V_th_) negatively (Figure 20d,e), modulating spike frequency outputs (Figure 20f). Salt detection utilizes sodium ionophore‐modified electrodes, with spike frequency scaling linearly with Na⁺ concentration (0g).^[^ ^227^ ^]^ Yang et al. constructed a complete artificial neuromorphic taste system by integrating gel sensors, SnO_2_ nanowire synapses, and effector execution units (Figure 20h). Synaptic conductance was modulated by ion migration (Figure 20i), enabling the system to achieve a shorter response delay (<1 s), a longer gustatory memory retention (>2 h), and a wide concentration detection range (0.02–6 wt.%). Notably, SnO_2_ nanowire‐based artificial synapses successfully encode taste information at an ultralow operating voltage (1 mV), surpassing biological counterparts by orders of magnitude (Figure 20j,k). This engineered gustatory system overcomes conventional limitations in taste sensing implementation, establishing a novel pathway for dietary health monitoring applications through its biomimetic signal transduction and adaptive learning capabilities.^[^ ^221^ ^]^

### 4.5 Artificial Olfactory Nervous System

In contrast to gustation, the mammalian olfactory system enables higher‐dimensional sensory perception through combinatorial coding of volatile organic compounds.

#### 4.5.1 Biological Mechanisms of Olfaction

**Figure 21:**
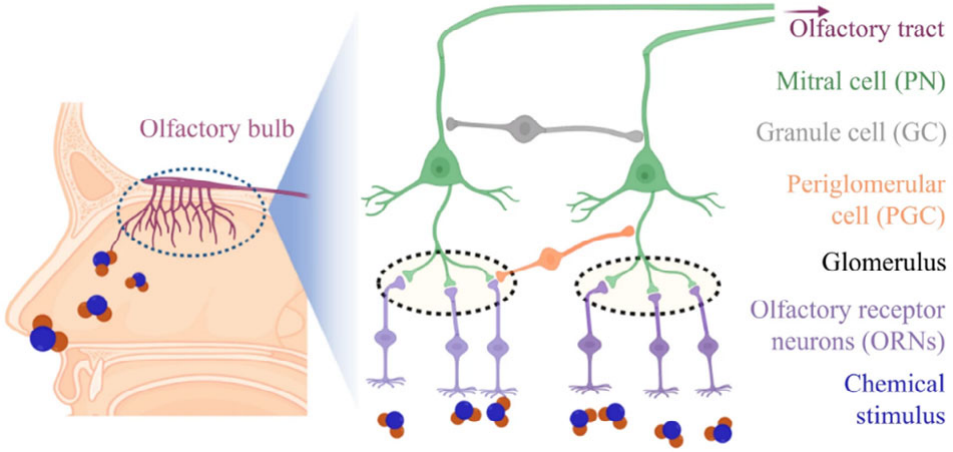
Schematic of the human olfactory system.^[^ ^228^ ^]^ Copyright 2023, The Author(s).

The human nose contains ≈400 types of receptors. Despite this limited receptor diversity, it can distinguish a vast number of odors through combinatorial coding mechanisms, enabling the detection of over 10 000 distinct odors. There are two primary pathways: the orthonasal pathway and the retronasal pathway. The orthonasal pathway involves environmental odor molecules ascending through the olfactory cleft during inhalation, reaching olfactory neurons at the nasal cavity terminus to induce odor perception. The retronasal pathway occurs during swallowing or exhalation, where odorants travel back through the nasopharyngeal passage connecting the nasal and oral cavities, subsequently contacting the olfactory epithelium at the nasal cavity terminus to elicit olfactory experiences (**Figure** **21**).^[^ ^228^ ^]^

#### 4.5.2 Bioinspired Olfactory Sensor

Inspired by biological olfactory processing mechanisms, electronic noses have been developed for odor detection. Traditional olfactory sensors suffer from bulky size, limited portability, and compromised reliability due to rapid chemical composition changes in target gases. Consequently, various novel gas‐sensitive detection devices have been explored. Current gas sensors can be categorized by signal transduction principles into electrochemical, optical, acoustic, thermal, and semiconductor‐based types.^[^ ^229^ ^]^ Semiconductor‐based sensors are widely investigated for their simplicity, portability, compatibility with standard electronics, continuous monitoring capability, and wireless transmission potential. The sensing materials include metal oxide semiconductors (MOS),^[^ ^230^, ^231^ ^]^ conductive polymers,^[^ ^232^, ^233^ ^]^ carbon nanotubes (CNTs),^[^ ^234^, ^235^ ^]^ and 2D materials.^[^ ^236^ ^]^ Furthermore, researchers are advancing the integration of large‐scale gas‐sensing devices into neuromorphic olfactory systems to emulate biological odor perception and processing.

#### 4.5.3 Artificial Olfactory Nervous System

**Figure 22:**
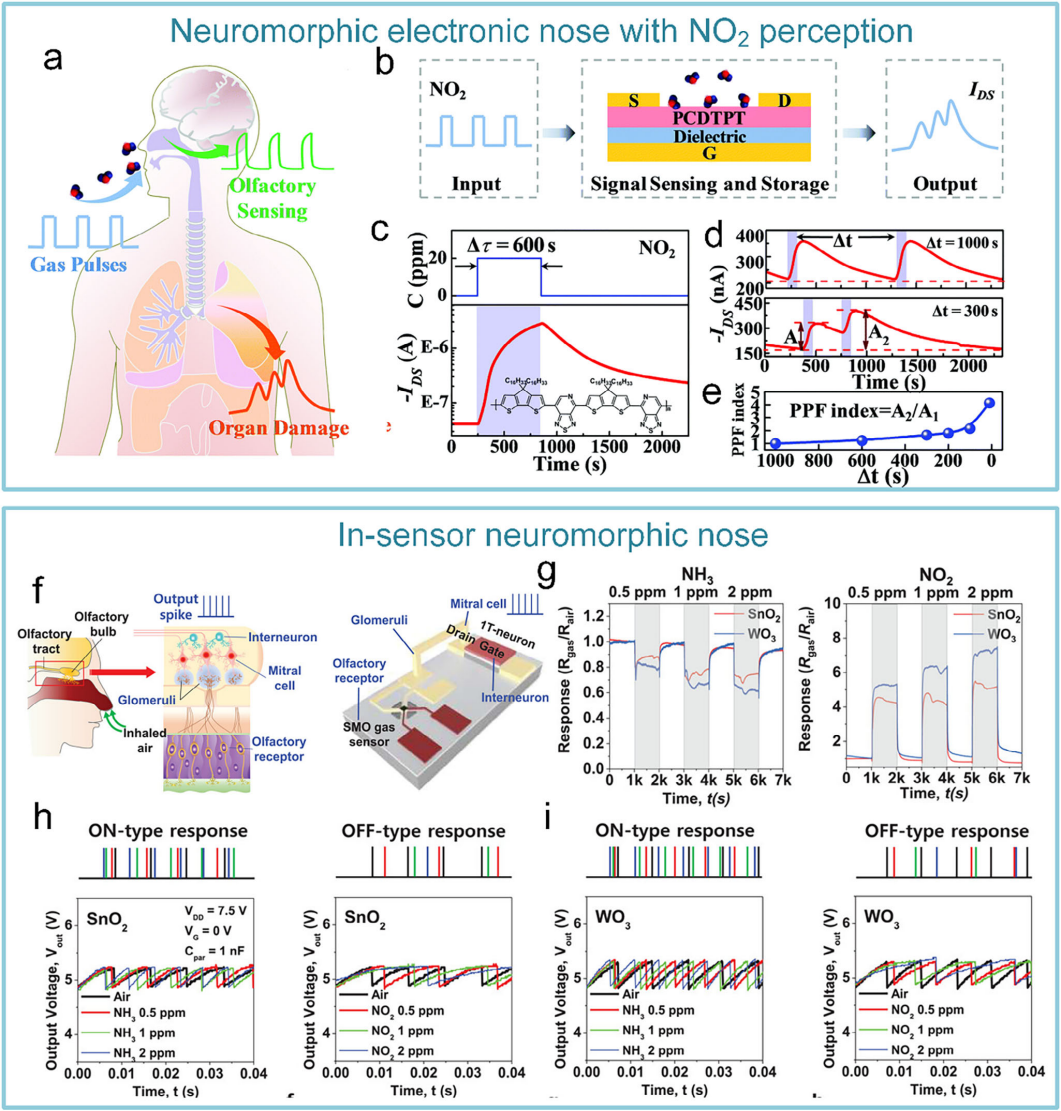
Artificial synaptic device‐based olfactory systems. a) Schematic of harmful gas effects on human olfactory receptors and organ systems. b) Operational model of the neuromorphic synaptic device. c) Response characteristics of the synaptic device under 20 ppm NO_2_ exposure. d) Real‐time current modulation under dual 20 ppm NO_2_ pulses with varying inter‐pulse intervals. e) PPF index at different pulse interval times.^[^ ^237^ ^]^ Copyright 2019, Royal Society of Chemistry. f) Comparative schematics of biological and artificial olfactory pathways. g) Dynamic responses of SnO_2_/WO_3_ sensors to varying NH_3_/NO_2_ concentrations. h) NH_3_/NO_2_ concentration‐dependent response profiles of SnO_2_‐based artificial olfactory neurons. i) Gas discrimination characteristics of WO_3_‐based artificial olfactory neurons across concentration gradients.^[^ ^238^ ^]^ 2022 The Authors. Advanced Science published by Wiley‐VCH GmbH.

By mimicking the way of biological olfactory systems process odors, Song et al. developed a neuromorphic synaptic device with NO_2_ detection and memory functions using solution‐processed poly[4‐(4,4‐dihexadecyl‐4H‐cyclopenta[1,2‐b:5,4‐b']dithiophen‐2‐yl)‐alt‐(1,2,5] thiadiazolo [3,4‐c]pyridine] (PCDTPT) thin films (**Figure** **22**a,b).^[^ ^237^ ^]^ The PCDTPT‐based device can sense and generate a drain–source current (ID_S_) with NO_2_ as the input pulse; the device can be used to detect the leakage of harmful gases at room temperature (Figure 22c). Moreover, the organic semiconductor channel exhibits gas memory effects through reversible adsorption/desorption cycles (Figure 22d,e). The test successfully simulated the organ damage caused by inhalation during hazardous gas leakage incidents. Inspired by multilevel biological olfactory neurons (olfactory receptors, mitral valve cells, glomerular cells, and neurons in the olfactory bulb), Han et al. developed an olfactory neuron composed of a semiconductor metal oxide gas sensor (SnO_2_/WO_3_) and a metal oxide‐semiconductor synaptic device (Figure 22f).^[^ ^238^ ^]^ The SnO_2_/WO_3_ gas sensors were fabricated by the glancing‐angle deposition method based on radio frequency sputtering, and Au nanoparticles were added to enhance the sensing effect. The resistance of the metal oxide was modulated through surface electron depletion layer changes, reductive gases decrease resistance, while oxidative gases increase resistance (Figure 22g). The electrical characteristics of the artificial olfactory neurons based on SnO_2_ and WO_3_ gas sensors under the action of different concentrations of NH_3_ and NO_2_ were tested in detail (Figure 22h,i). The system achieves gas discrimination without complex logic circuits, mimicking olfactory bulb signal processing.

**Figure 23:**
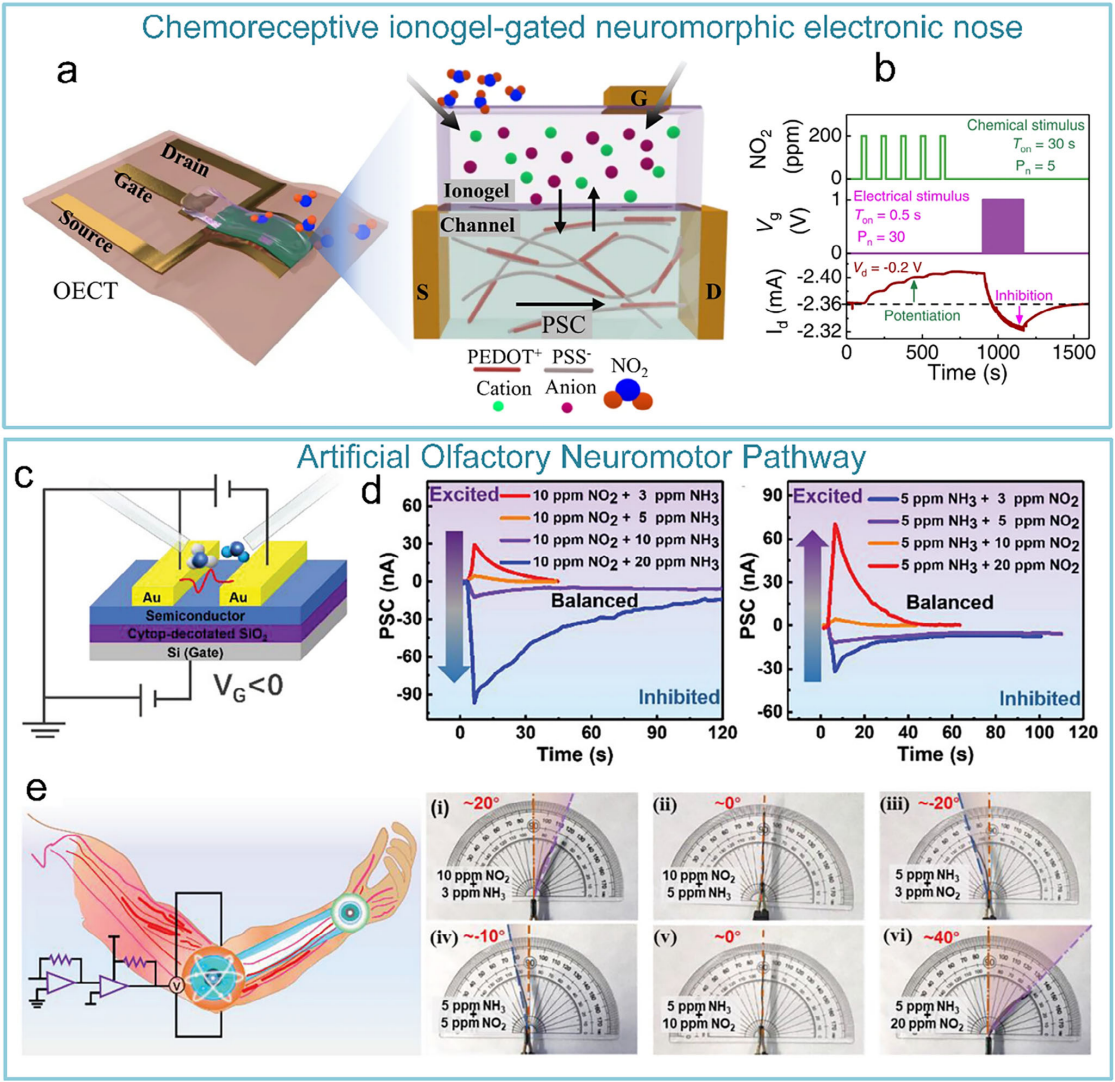
a) Flexible olfactory neuromorphic device and corresponding mechanism diagram. b) excitatory inhibition function graph.^[^ ^228^ ^]^ Copyright 2023, The Author(s). c) Schematic diagram of olfactory neuromorphic devices. d) Response characteristics under different concentrations of NO_2_ and NH_3_. e) Deflection angles of artificial muscles under different gas concentrations.^[^ ^239^ ^]^ Copyright 2024 Wiley‐VCH GmbH.

**Figure 24:**
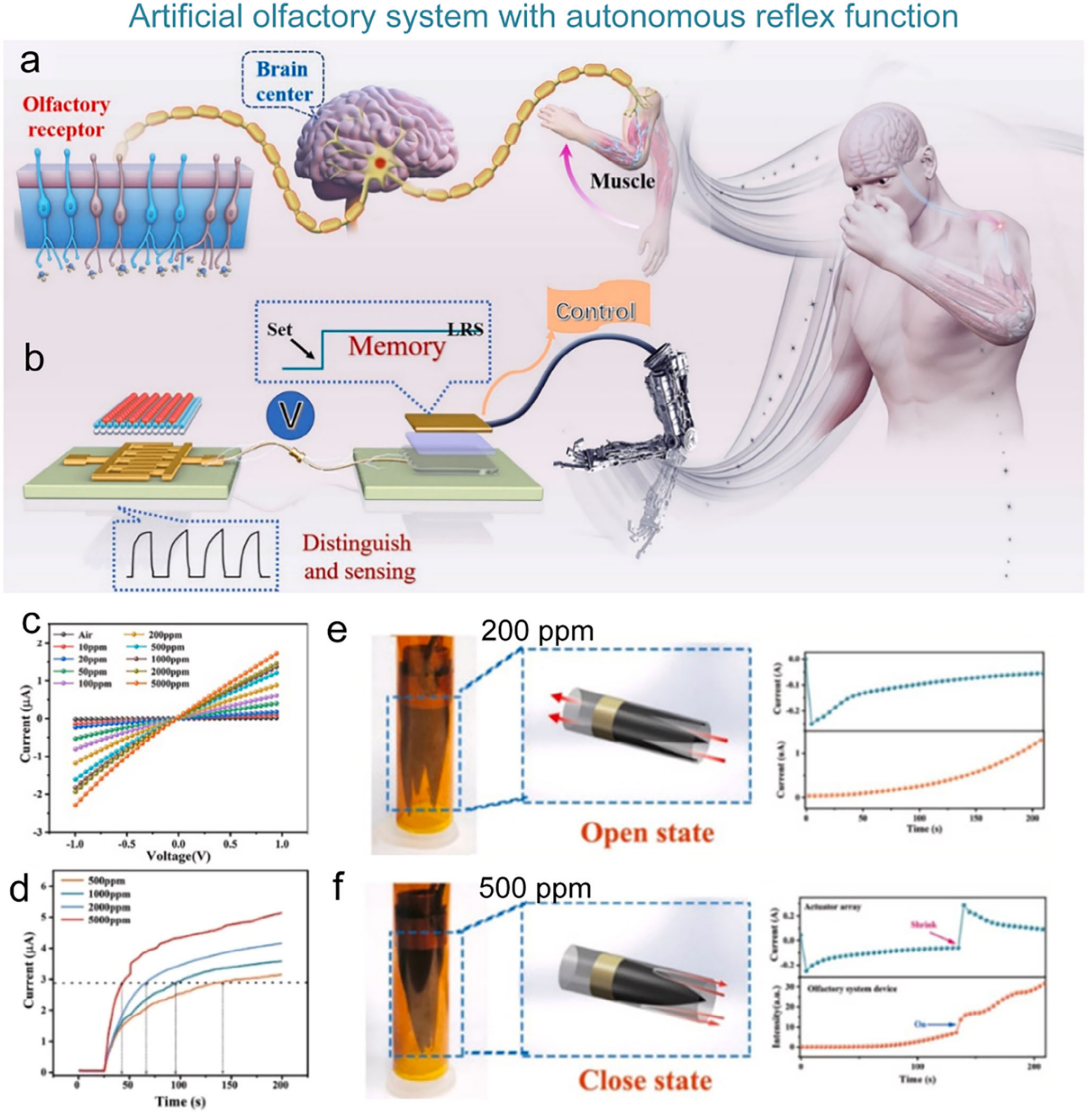
Artificial Olfactory System Based on Sr‐ZnO Gas Sensor and Memristor.a) Schematic of the human olfactory system, comprising three key components: olfactory receptors, central processing centers, and effector muscles. b) Gas sensor responding to stimuli through voltage division modulation. c) Current–voltage (I–V) characteristics of the gas sensor under varying NH_3_ concentrations. d) Memristor current response modulation across NH_3_ concentration gradients. e) Transient current dynamics under low NH_3_ exposure (200 ppm). f) High‐concentration NH_3_ response (500 ppm) with saturation behavior.^[^ ^240^ ^]^ Copyright 2021 Elsevier Ltd. All rights reserved.

Chouhdry et al. developed a flexible artificial synaptic neuron on PI substrates, emulating excitatory/inhibitory neurotransmitter dynamics (**Figure** **23**a).^[^ ^228^ ^]^ When the device is exposed to an environment filled with NO_2_, NO_2_ induces negative gating in the electrolyte layer, increasing hole conductivity in the p‐type organic channel. Conversely, positive gate voltages suppress channel current, enabling bidirectional synaptic modulation within a single device (Figure 23b). The system provides a hardware foundation for neuromorphic olfaction. Wu et al. fabricated a gas‐sensitive synaptic device using poly (diketopyrrolopyrrole‐selenophene) (PTDPPSe‐5Si) as the channel layer (Figure 23c). The device mimics biological excitation‐inhibition balance regulation through electron donor/acceptor gas‐induced carrier trapping/release mechanisms (Figure 23d). The olfactory neural pathway was constructed by integrating synaptic devices with artificial muscles; the system establishes a gas stimulus‐to‐motor reflex mapping via controlled bending actuation (Figure 23e). Furthermore, this architecture achieves 97.3% accuracy in identifying eight laboratory gases, demonstrating adaptive olfactory‐motor coordination.^[^ ^239^ ^]^ Gao et al. developed an artificial olfactory neural system that integrates gas sensing, information processing, and bioinspired avoidance reflexes (**Figure** **24**a,b).^[^ ^240^ ^]^ The system can detect NH_3_ through chemiresistive modulation, real‐time memory storage via synaptic device conductance states, and self‐protective “nose‐covering” behavior emulation by triggering actuator bending to block gas inflow (Figure 24c–f). Remarkably, these perception and reflex functions are achieved without complex signal processing circuits. This innovative approach provides valuable insights for olfactory sensory system simulation research.

### 4.6 Artificial Multimodal Nervous System

In the previous section, we have summarized the nervous systems of the different senses, including the biological mechanisms and the corresponding artificial nervous system study. Each sensory nervous system sustains a variety of human needs, yet the nervous system of human is highly complex, it can process information from a variety of sensory modes to produce accurate behaviors. Exhibiting remarkable sophistication in processing multimodal sensory inputs to generate coordinated behavioral outputs capability termed multisensory integration. This complex process plays an essential role in perceptual synthesis, cognitive decision‐making, and adaptive behaviors. For instance, our brain achieves comprehensive environmental perception by simultaneously processing tactile feedback from the skin, visual scenes through ocular input, auditory signatures of object motion, olfactory cues via nasal chemoreception, and gustatory stimuli detected by taste buds. Developing multifunctional artificial neural systems capable of integrating multimodal plasticity, memory capacity, and supervised learning capabilities represents an imperative frontier in neuromorphic research.

**Figure 25:**
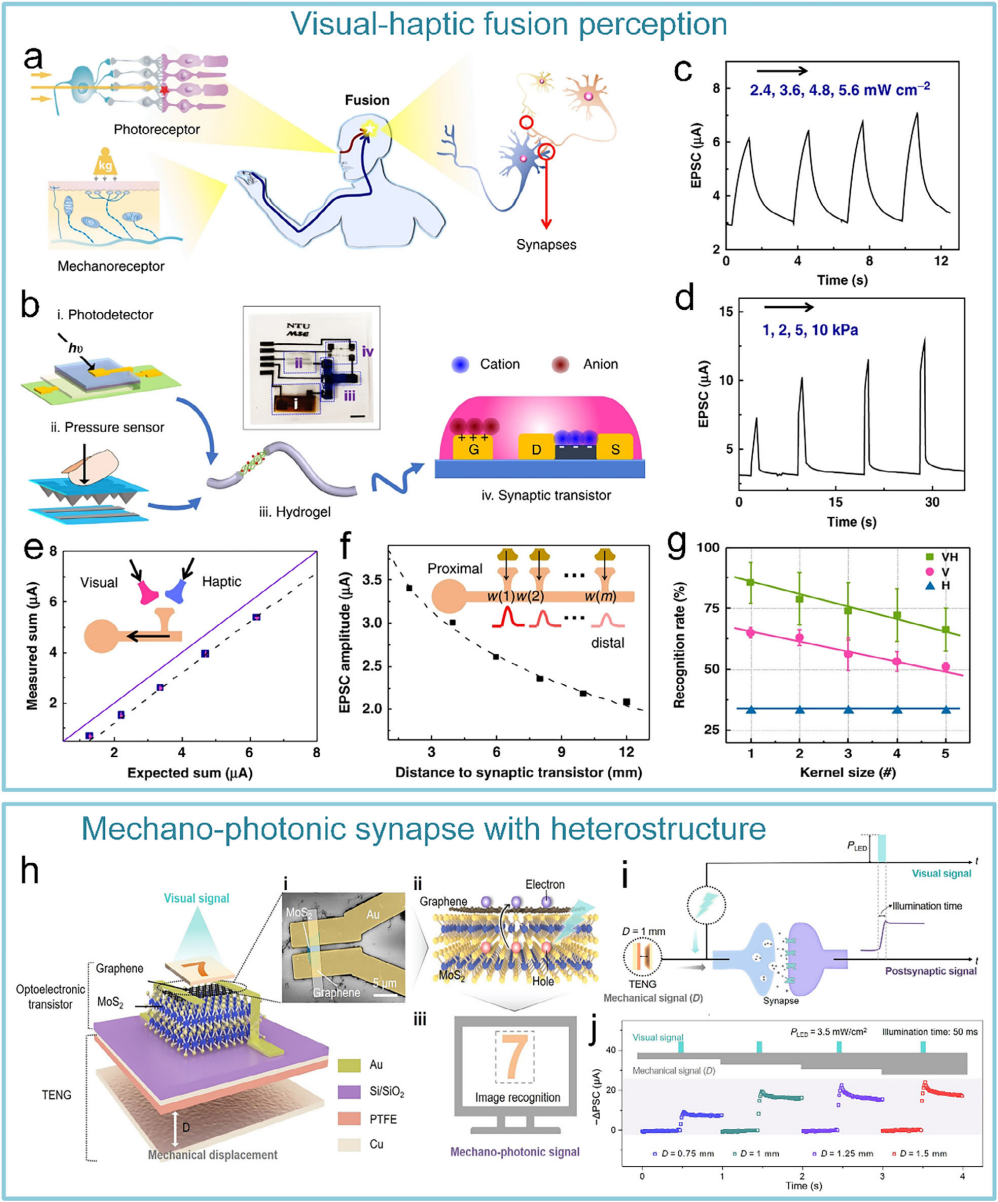
Bimodal mechano–optical artificial neural systems. a) Schematic of visuotactile integration pathways in human sensory processing. b) Architecture of the artificial visuotactile neural system. c) Synaptic device response under varying light intensities. d) Pressure‐dependent synaptic modulation characteristics. e) Fusion output from equidistant multimodal inputs. f) Distance‐dependent response attenuation; g) Recognition accuracy comparison: unimodal versus bimodal fusion.^[^ ^241^ ^]^ Copyright 2020, The Author(s). h) Mechanophotonic artificial synapse integrating TENG and graphene/MoS_2_ heterostructure. i) Synergistic modulation mechanism of mechanical displacement and optical signals. j) Synergistic current response under illumination and variable TENG gaps (0.75–1.5 mm) at V_D_ = 1 V.^[^ ^242^ ^]^ Copyright 2021, The American Association for the Advancement of Science.

Inspired by the biological complex perception function, by integrating a perovskite‐based photodetector, a piezoresistive pressure sensor, PVA hydrogel ionic cables, and a synaptic device, Wan et al. constructed a bimodal artificial neuron system (**Figure** **25**a,b).^[^ ^241^ ^]^ This structure simulates the biological afferent nerve. It was realized that multi‐sensory integration through parallel processing of optical and tactile stimuli has a stable response to different intensities of light stimulation and different sizes of force stimulation (Figure 25c,d). Applied to robotic control, the system demonstrates enhanced object recognition accuracy through visuotactile fusion, enabling texture discrimination and shape identification (Figure 25e–g). Yu et al. engineered a mechano–photonic bimodal synapse device integrating graphene/MoS_2_ optoelectronic transistors with triboelectric TENG (Figure 25h).^[^ ^242^ ^]^ Synaptic plasticity is synergistically regulated by mechanical strain and optical pulses (Figure 25i,j). The device demonstrates spatiotemporal learning rules with STDP similarity to biological counterparts, enabling energy‐efficient multimodal memory consolidation.

**Figure 26:**
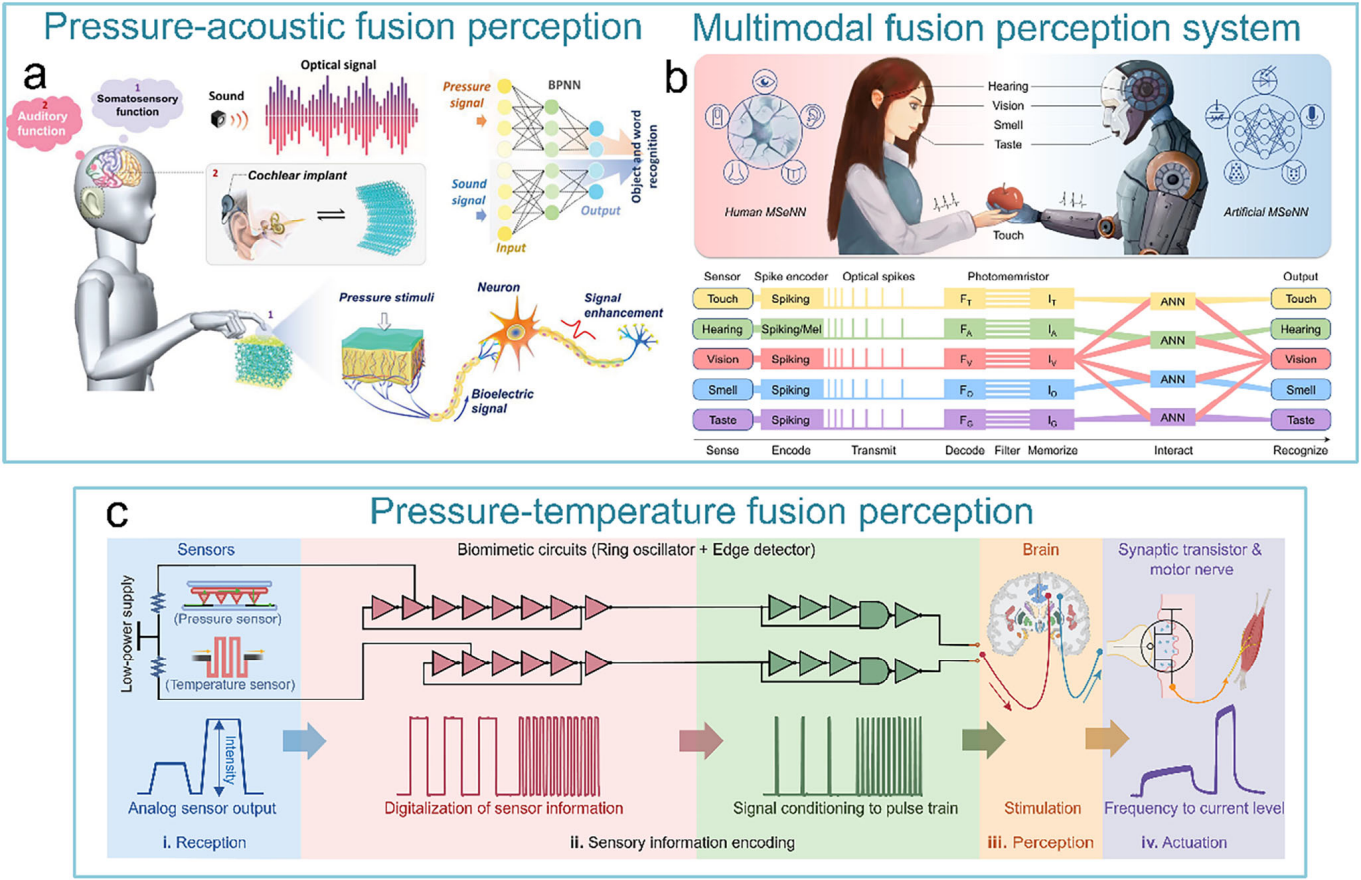
Bimodal artificial nervous systems. a) Auditory‐tactile bimodal artificial nerve.^[^ ^243^ ^]^ Copyright 2023 Wiley‐VCH GmbH. b) Multimodal sensory fusion system and corresponding signal conversion process.^[^ ^244^ ^]^ Copyright 2021, The Author(s). c) Pressure–temperature bimodal perception system.^[^ ^15^ ^]^ Copyright 2023, The American Association for the Advancement of Science.

Xu et al. developed a bimodal sensory system integrating tactile and auditory perception functions (**Figure** **26**a). By combining hydrogen‐bonded organic frameworks with melamine foam, the system has ultralow detection thresholds (13.33 × 10^−3^ Pa for pressure, 0.36 dB for sound) and exceptional sensitivities (132.02 kPa^−1^, 1.648 × 10⁶ cps·Pa^−1^·cm^−2^). This dual‐modal architecture enables high‐accuracy recognition of nine object types through spatiotemporal signal fusion.^[^ ^243^ ^]^ Tan et al. engineered a pentamodal neural network emulating tactile, visual, auditory, gustatory, and olfactory processing (Figure 26b). Sensory transducers (photodetectors, piezoresistors, acoustic sensors, and chemo‐resistive taste/odor receptors) convert external stimuli into electrical potentials. Frequency encoders transform these potentials into spike trains, which are decoded and filtered by photoresistive memory modules. The processed signals undergo cross‐modal integration in an artificial neural network, achieving multisensory imagination accuracy for stimulus combinations.^[^ ^244^ ^]^ Wang et al. developed a biohybrid sensorimotor system that establishes a closed‐loop artificial neural pathway integrating soft electronic skin with rodent neural circuits (Figure 26c). Electronic skin capable of detecting pressures encodes tactile stimuli through ring oscillators and edge detectors, converting mechanical inputs into frequency‐based neural signals. These encoded signals are transmitted to the rat's somatosensory cortex, amplifying cortical outputs that then drive synaptic transistors, which generate graded stimulation pulses for sciatic nerve activation, achieving precise hindlimb flexion control. This biomimetic architecture effectively replicated natural sensorimotor integration. The system can enable real‐time closed‐loop interaction between artificial tactile encoding and biological motor execution.^[^ ^15^ ^]^

## 5 Application Fields of Artificial Nervous System

Artificial neuromorphic devices and systems demonstrate extensive applicability in neuromorphic image recognition, neuromorphic prosthesis, soft robotics, and neuromorphic biohybrid systems.

### 5.1 Neuromorphic Image Recognition

Image recognition represents a significant application in artificial neuromorphic systems, whose core principle involves mimicking biological learning and information processing mechanisms to evaluate recognition accuracy through multiple iterations of dataset training.

**Figure 27:**
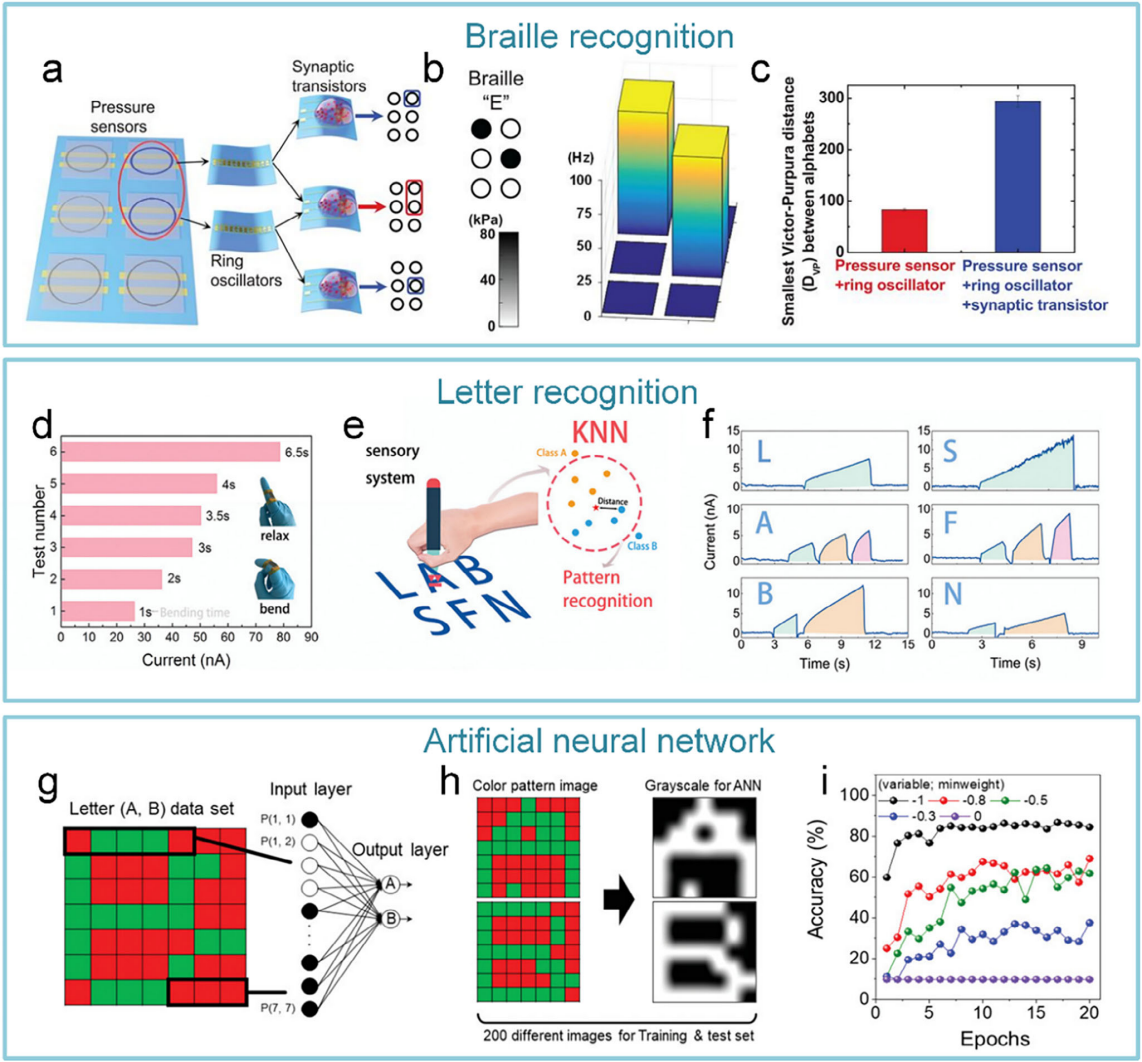
a) Schematic architecture of the Braille‐reading neuromorphic module. b) Presynaptic spike frequency modulation characteristics under mechanical stimulation of the pressure sensor array (left), with corresponding postsynaptic current in a single‐pixel synaptic transistor (right). c) Minimum Victor‐Purpura distance between EPSC of different letters.^[^ ^14^ ^]^ Copyright 2018, The American Association for the Advancement of Science. d) Time‐dependent current responses of the phalange‐mounted flexible sensor during 1–6.5 s flexion cycles. e) Operational schematic of the intelligent character‐writing interface. f) Distinctive current signatures corresponding to alphabetical character inputs.^[^ ^245^ ^]^ Copyright 2019 WILEY‐VCH Verlag GmbH & Co. KGaA, Weinheim. g) ANN implementation framework for optical pattern recognition. h) Training image set containing characters “A” and “B” with progressive distortion levels. i) Performance metrics demonstrating 86.82% MNIST recognition accuracy across 20 training epochs.^[^ ^246^ ^]^ Copyright 2021 Wiley‐VCH GmbH.

Kim et al. proposed a braille character recognition system utilizing a pyramid‐architected piezoresistive sensor array (3 × 2) to emulate the distributed mechanoreception of biological tactile systems.^[^ ^14^ ^]^ Pressure signals were transduced into pulse stimuli via ring oscillators, while the synaptic device integrated multi‐channel input signals (**Figure** **27**a,b). Following synaptic signal integration, the device accurately recognized distinct braille characters (Figure 27c). Additionally, this flexible organic electronic device facilitates dynamic pressure detection and orientation sensing. Zhang et al. developed an artificial haptic nervous system through integration of a piezoresistive sensor and a synaptic transistor.^[^ ^245^ ^]^ By modulating the sensor's applied pressure level, frequency, duration, and speed, the device's postsynaptic current was regulated to identify corresponding tactile signals. Mounted on a finger joint, the system exhibited time‐dependent current responses under flexion. The platform demonstrates broad applicability in human–computer interaction interfaces for motion sensing and gesture control (Figure 27d). When connecting an intelligent stylus to the sensing element, distinct output current waveforms were generated during letter‐writing tasks, where peak currents served as discriminative features for stroke duration analysis (Figure 27e,f). Kwon et al. developed a photonic synaptic pixel circuit comprising an IGZO/CdS heterojunction synaptic device and a CdSe photoresistor‐photovoltaic voltage divider.^[^ ^246^ ^]^ The system achieves bidirectional synaptic LTP/LTD modulation under multi‐wavelength optical stimulation, using green light (525 nm for excitation) and red light (620 nm for inhibition). In letter recognition tasks (“A” vs “B”), green light activated target pixels, while red light suppressed background pixels. Postsynaptic current (PSC) mapping generated grayscale images with reduced training errors and a recognition accuracy of 97.65%, outperforming unidirectional modulation. Scaling to a 20 × 20 pixel array and simulating an artificial neural network, the system achieved 86.82% accuracy on the Modified National Institute of Standards and Technology database (MNIST) handwritten digit recognition (Figure 27g–i).

### 5.2 Neuromorphic Prosthesis

**Figure 28:**
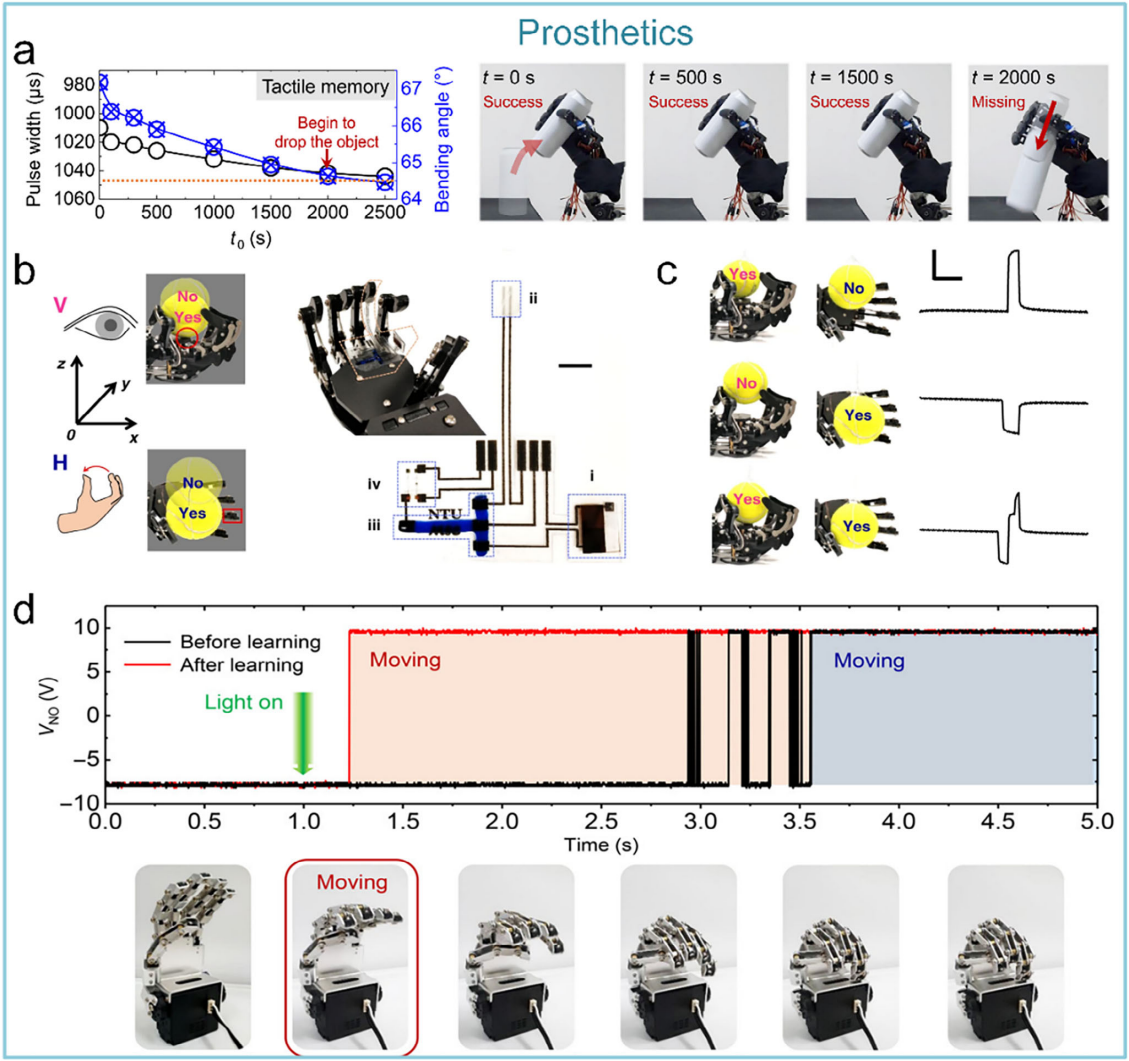
a) Dynamic correlation between pulse width modulation and joint flexion angles (left), with sequential snapshots demonstrating repetitive grasping motions of the neuro‐inspired robotic end‐effector (right).^[^ ^16^ ^]^ Copyright 2023, The American Association for the Advancement of Science. b) System architecture of the BASE integrated on robotic manipulator surfaces (left), accompanied by its epidermal implementation schematic (right). c) Quantified ΔEPSC (excitatory postsynaptic current) variations reflecting spatial resolution of spherical object localization through BASE sensors.^[^ ^241^ ^]^ Copyright 2020, The Author(s). d) Real‐time robotic hand manipulation sequences (upper panel) synchronized with ANN‐triggered voltage signals post‐learning optimization (lower panel), highlighting the system's reliability in conscious‐level motor emulation.^[^ ^198^ ^]^ Copyright 2021, The American Association for the Advancement of Science.

Current biomimetic haptic systems encounter challenges in transient tactile memory maintenance and complex circuit integration. To address these limitations, Kweon et al. developed a neuron‐inspired monolithic artificial tactile neuron that integrates tactile signaling and memory functions through stress‐triggered ion release within a gel.^[^ ^16^ ^]^ This design enables synergistic enhancement of tactile signal processing and memory consolidation. Integrated into a robotic hand system, this neuro‐inspired neuron demonstrated reliable grasping operations, maintaining stable performance for 2000 s without control errors caused by signal attenuation (**Figure** **28**a). Furthermore, Wan et al. engineered a bimodal artificial sensory neuron (BASE) combining visual (perovskite photodetector) and tactile (pressure sensor) signals, transmitted via ionic cables to synaptic transistors for skeletal muscle tube control and multi‐transparency pattern recognition.^[^ ^241^ ^]^ The fusion of visual‐tactile modalities enhanced spatial perception, improving decision‐making accuracy in robotic grasping tasks. Integration with C2C12 skeletal muscle tubes validated bioelectronic interfacing by electrically driving muscle contraction (Figure 28b,c). Kim et al. designed a tactile‐responsive system incorporating quantum dot photodetectors, electric double‐layer synaptic transistors, CMOS neuron circuits, and robotic hands to mimic human unconditioned reflex.^[^ ^198^ ^]^ By optimizing response times through repeated learning and multimodal signal processing (optical, electrical, ionic), this system replicated complete neural pathways from perception to action. Synaptic plasticity (LTP/LTD) emulation reduced robotic hand latency from 2.56 to 0.23 s (Figure 28d).

### 5.3 Neuromorphic Soft Robotics

**Figure 29:**
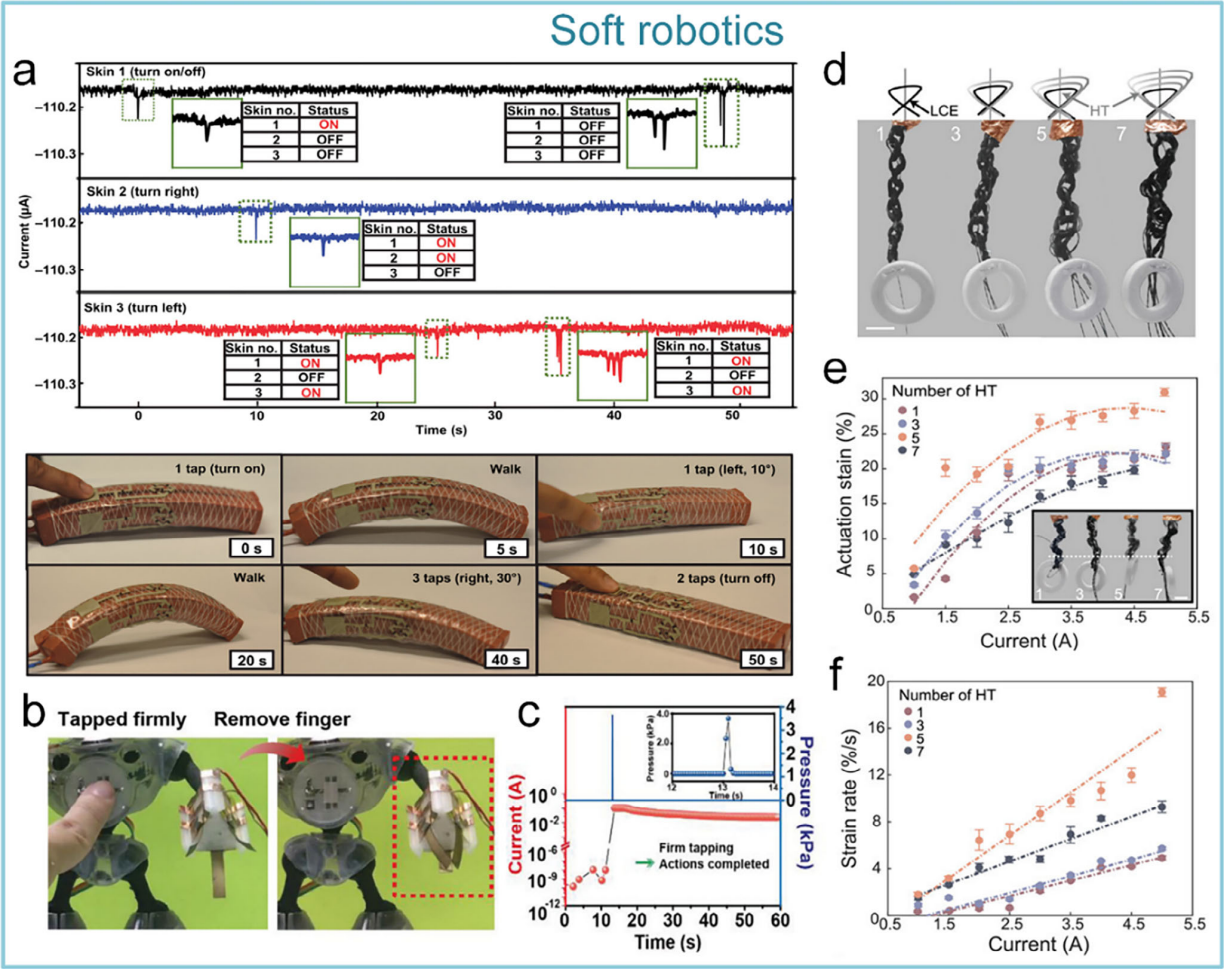
a) Spatiotemporal mapping of EPSC across dorsal, lateral, and ventral epidermal regions during 50‐s locomotion cycles (left), with corresponding temporal profiles of soft neuromorphic robot kinematics (right).^[^ ^147^ ^]^ Copyright 2019, The American Association for the Advancement of Science. b) Robotic hand reflex dynamics under finger percussion stimuli. c) Transient current modulations in electrochemical actuators correlating with applied pressure intensity.^[^ ^144^ ^]^ Copyright 2019 WILEY‐VCH Verlag GmbH & Co. KGaA, Weinheim. d) Structural evolution of artificial muscle bundles: schematic illustrations (top) and photographic documentation (bottom) of knotting configurations with increasing filament multiplicity. e) Comparison of actuation strain versus input current density across multifilament assemblies. f) Correlation between strain rate enhancement and heating wire population in optimized muscle architectures.^[^ ^247^ ^]^ Copyright 2024 Wiley‐VCH GmbH.

Shim et al. developed a stretchable synaptic transistor using all‐rubber materials (stretchable semiconductors, conductors, and gels), which maintained stable synaptic properties under 50% strain.^[^ ^147^ ^]^ A stretchable sensory skin array was constructed by integrating mechanoreceptors with synaptic transistors, enabling tactile‐to‐postsynaptic potential mapping. Leveraging synaptic memory‐encoded signals, this system controlled soft robots to perform adaptive locomotion along complex paths, pioneering neural functional integration in soft robotics (**Figure** **29**a). He et al. mimicked the biological “all‐or‐none” reflex arc by combining pressure sensors, metal–organic framework‐based threshold control units, and electrochemical actuators.^[^ ^144^ ^]^ Integrated into robotic systems, this design enabled infant‐like grasp reflexes in robotic fingers, demonstrating potential for soft robotics and neuroprosthetics (Figure 29b,c). Chen et al. achieved high‐frequency, large‐stroke deep‐water actuation through 3D‐printed liquid crystal elastomer fibers interwoven with heating wires into knotted bundle structures.^[^ ^247^ ^]^ This system realized reliable deep‐sea operation (3000 m) with pressure resistance surpassing conventional dielectric elastomers, retaining 20% strain even under partial fiber fracture. By braiding multiple heating wires into square‐knotted bundles, the actuator achieved exceptional strain (32%) and strain rates (19% s^−1^) under increased power input (Figure 29d–f).

### 5.4 Neuromorphic Biohybrid System

Connecting artificial nervous systems with biological nerves and muscles allows the construction of neuromorphic hybrid biological systems, aiming to validate the potential of artificial nervous systems in repairing damaged nerves and remodeling sensorimotor functions.

**Figure 30:**
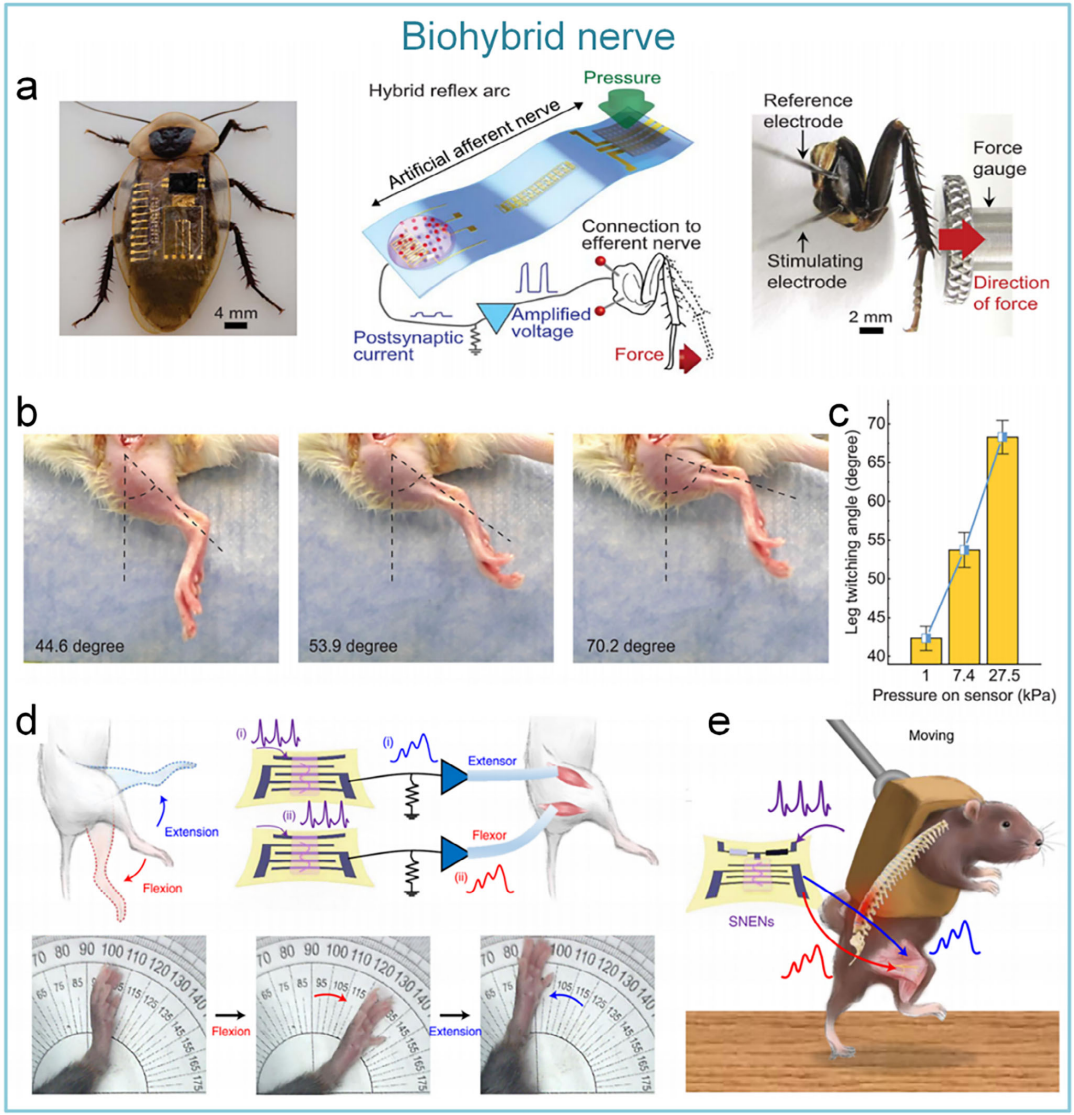
a) Hybrid system architecture: macroscopic view (left) and circuit schematic (right) of the artificial afferent‐biological efferent neural loop integrated with specimens.^[^ ^14^ ^]^ Copyright 2018, The American Association for the Advancement of Science. b) Electrophysiological validation of the neuroprosthetic interface‐ photographic documentation of hindlimb withdrawal reflex in murine models with corresponding angular displacement measurements. c) The correlation between leg twitch angle and applied pressure.^[^ ^15^ ^]^ Copyright 2023, The American Association for the Advancement of Science. d) Bipedal locomotion rehabilitation framework: implantable device schematic and sequential motion capture of knee flexion‐extension cycles. e) Diagram of a mouse using SNEN to restore autonomic motor function.^[^ ^23^ ^]^ Copyright 2022, The Author(s).

By connecting artificial afferent nerves to the legs of cockroaches, Kim et al. built the first neuromorphic hybrid system based on artificial nerves to control biological muscle movement. The pressure sensor converts the pressure that comes from different spatio – temporal into electrical signals to realize the perception of external information. The information coding process is further carried out by a ring oscillator to transmit the external information in the form of frequency pulses. Then, the neuromorphic device integrates different pulse information based on SFDP characteristics and outputs EPSC. Finally, EPSC was amplified to realize a real‐time swing of the cockroach's legs under the control of external pressure. In this process, the external force showed a positive correlation with the angle swing and the output force of the cockroach (**Figure** **30**a).^[^ ^14^ ^]^ Wang et al. connected the artificial nervous system with brain regions and sciatic nerves of mammals (rats) to construct a sensor‐motor hybrid system with multimodal sensing, neuromorphic peak signal processing, and closed‐loop drive capabilities. The sensor in the system can realize the perception of pressure and temperature, the ring oscillator and the edge detector can realize the accurate encoding of electrical signals, the encoded information is transmitted to the rat sensory cortex, the information from the motor cortex is applied to the neuromorphic device, and after integration and amplification processing, the real‐time driving of the rat leg is realized (Figure 30b,c).^[^ ^15^ ^]^ Lee et al. connected a stretchable artificial nervous system with a rat leg to construct a closed‐loop feedback‐regulated hybrid system. The system can withstand 10^3^ cycles of stretching at a large strain range of 100%. And the leg swing corresponds in real time to the applied stimulus. Furthermore, the system was applied to rats with spinal cord injuries, and the rats can kick a ball, walk, or run. And the system only needs 1/150 of the power consumption of a traditional microcontroller. It provides a new idea to help the disabled organism reshape the sensory movement (Figure 30d,e).^[^ ^23^ ^]^

## 6 Conclusion and Outlook

**Figure 31:**
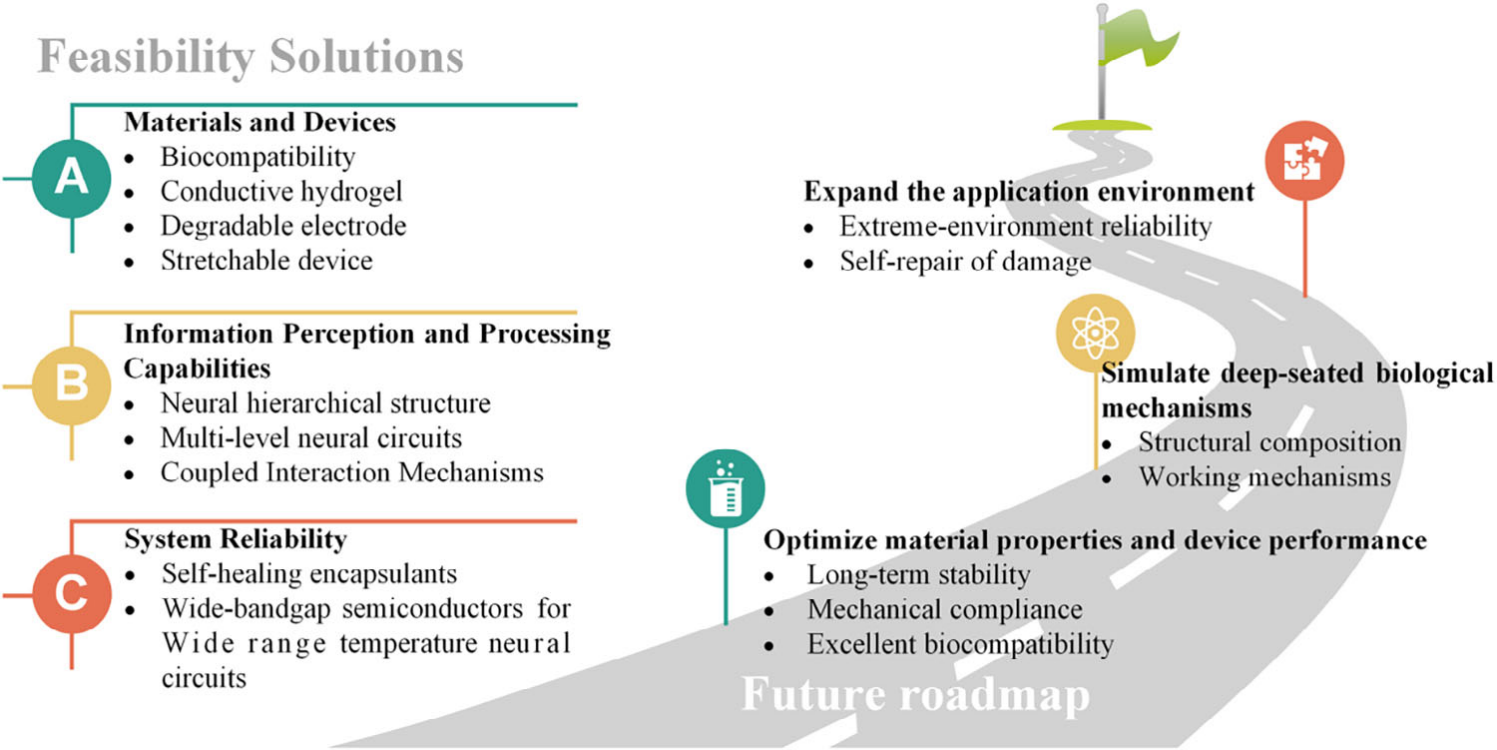
Future roadmap and key challenges of the artificial nervous system.

This review provides a comprehensive overview of artificial nervous systems’ recent advancements, which include bio‐inspired tactile, visual, olfactory, gustatory, auditory, and multisensory systems. According to the composition of the system, we have successively discussed the materials and structures of different modal sensors (information perception units), the structural, common materials, and processing techniques of synaptic devices (information processing units), and the construction and applications of complete artificial nervous systems (image recognition/human–machine interaction/biological interfaces). Research teams have constructed artificial nervous systems with multimodal perception, neural signal processing, and driving reflex functions by integrating multimodal sensors, neuromorphic synaptic devices, and effector units. The real‐time driving of mechanical hands, artificial muscles, and biological limbs by artificial nervous systems proves the potential application prospects in the field of prosthetic limb control and neural repair. However, to fully exploit the potential of these devices and ultimately promote their practical applications, there are still many technical challenges. This section will briefly categorize and introduce the key challenges currently faced (**Figure** **31**).

### 6.1 Challenges

Although artificial nervous systems have made remarkable progress, at the same time, we can also see that previous studies focused on the realization of the perception and driving functions of the artificial nervous system. Many aspects, e.g., the material properties, biocompatibility, and the applicable environment, of the system require further investigation.

In the previous research, under ideal conditions (room temperature), the sensor, synaptic device, and effector unit were simply connected, and the output signal was directly amplified to drive the effector unit. Once the perceptual unit of the artificial nervous system is damaged or the environmental temperature drops, its perceptual performance will gradually be lost, causing a mismatch between perceptual information and reflex activities. The unique advantage of biological systems lies in the strong adaptability (e.g., self‐regulation over a wide temperature range, self‐repair of damage), and the signals are transmitted in the form of frequency pulses. In addition, conventional rigid interconnects cause significant mechanical mismatch against soft biological tissues, leading to implantation trauma and signal attenuation. Therefore, we can also see that the existing artificial neural systems exhibit critical limitations when benchmarked against biological counterparts.

#### 6.1.1 Simulate Deep‐Seated Biological Mechanisms

Although artificial nervous systems have made remarkable progress, at the same time, we can also see that previous research has mainly focused on the realization of the most fundamental perception and driving functions of artificial nervous systems. The sensor, synaptic device, and effector unit were simply connected through wires to form an artificial nervous system with a sequential operating system, and the output signal is directly amplified to drive the effector unit. The strong information perception and processing capabilities of the biological nervous system stem from its complex neural hierarchical structure and plastic processing mechanism. Therefore, compared with biological nervous systems, current research on artificial nervous systems is insufficient in terms of structural composition and working mechanisms. This insufficient exploration also restricts the further application and promotion of artificial nervous systems.

Therefore, it is necessary to integrate neuroscience and leverage the increasingly mature brain imaging techniques (e.g., electron microscopy, functional magnetic resonance imaging, and optogenetics) and the progress of brain–computer interfaces in recent years, establishing a detailed map of all‐round connections between the human and animal brains from the perspective of the neuronal level and neural signal cognition. From a single neuron to multilevel neural circuits, and ultimately introducing coupled interaction mechanisms in multilevel neural circuits. From the bottom up, comprehensively understand and simulate the complexity of the biological nervous system.

#### 6.1.2 Expand the Application Environment

Current research on artificial nervous systems mostly remains at the level of theoretical basis exploration. In previous studies, experiments were mostly conducted under ideal conditions in laboratories (room temperature and humidity). Once the perception units of the artificial nervous system are damaged or the environmental temperature drops, its perception performance will gradually decline or even be lost, resulting in a mismatch between perceived information and reflex activities. The unique advantage of biological systems lies in their strong adaptability (e.g., self‐regulation over a wide temperature range, self‐repair of damage). Therefore, a system with high reliability and long‐term stability is needed, especially in harsh environments, where rapid and efficient self‐healing is a significant challenge.

Therefore, it is necessary to combine materials science and introduce different bonds (reversible dynamic hydrogen bonds, coordination bonds, borate ester bonds, covalent disulfide bonds, etc.) into the flexible hydrogel‐based sensing unit to ensure its healing performance. And by constructing a multiple composite network system, introducing small‐sized nanoparticles as chemical crosslinking sites, and introducing electrolyte salts to form coordination bond effects, to enhance the applicability in complex environments.

#### 6.1.3 Optimize Material Properties and Device Performance

In artificial nervous systems, primary neural modules can be connected to deeper nervous systems to form complex neural architectures due to their excellent mechanical compliance (low modulus, high tensile strength, etc.). At present, the construction of artificial nervous systems is more about simply connecting different modules (sensors, synaptic devices, and effect units). More importantly, the traditional rigid interconnection method has a significant mechanical mismatch problem with the soft biological tissue (1 to 100 kPa), and it causes severe trauma during the process of implantation into the organism. Although flexible and stretchable materials have achieved certain development, there are still some deficiencies in terms of performance and durability. This leads to the function and diversity of stretchable devices being difficult to match the traditional silicon‐based devices.

Therefore, to address the aforementioned issues, it is essential to significantly enhance the performance of flexible electronic products and improve their stability and durability. This not only requires the development of new flexible materials, but also the design of multiple types of devices, the improvement of integrated processing techniques, and certain optimizations and upgrades to the current flexible electronic technology.

### 6.2 Perspectives

Based on the above, we can see that due to material defects, insufficient device performance, and the insufficient in‐depth bionic mechanism of the system, the further practical application of artificial nervous systems has been severely restricted.

To realize clinically viable artificial nervous systems with biological‐grade functionality, three hierarchical design principles must be synergistically addressed. First, synthetic high‐biomimetic materials. The foundation lies in developing biocompatible polymers exhibiting tissue‐mimetic mechanical compliance and self‐healing performance. Second, fabricating high‐performance devices with high environmental robustness. The sensing device still has a stable response in wide temperature environments; the information processing device has strong information integration and dynamic regulation functions. Thirdly, constructing a nervous system with advanced paradigms and adaptive control mechanisms. It should be a flexible and stretchable multimodal nervous system. This multiscale engineering framework bridges the critical gap between current rigid periprosthetic and biological systems, enabling next‐generation neural interfaces that adaptively coexist with living tissues while performing complex sensorimotor tasks under real‐world conditions.

Achieving these transformative capabilities requires the establishment of a convergence science framework that integrates microelectronics, advanced materials engineering, neuromorphic computing, and computational neuroscience. As artificial nervous systems evolve from rigid electromechanical to soft biologically symbiotic interfaces, they will witness the emergence of truly bio‐integrated neuromorphic technologies capable of seamless fusion with the human nervous system, ultimately bridging the boundary between biological and artificial nervous networks in clinical practice.

## Conflict of Interest

The authors declare no conflict of interest.
